# Molecular Underpinning of Treatment-Resistant Schizophrenia: A Putative Different Neurobiology from Treatment-Responsive Schizophrenia

**DOI:** 10.3390/ijms26178598

**Published:** 2025-09-04

**Authors:** Annarita Barone, Licia Vellucci, Mariateresa Ciccarelli, Marta Matrone, Giuseppe De Simone, Federica Iannotta, Felice Iasevoli, Andrea de Bartolomeis

**Affiliations:** 1 Section of Psychiatry, Laboratory of Translational and Molecular Psychiatry, Unit of Treatment-Resistant Psychosis, Department of Neuroscience, Reproductive Sciences and Dentistry, University Medical School of Naples “Federico II”, Via Pansini 5, 80131 Naples, Italy; 2 Department of Translational Medical Sciences, University of Naples “Federico II”, Via S. Pansini 5, 80131 Naples, Italy; 3 NESMOS (Neurosciences, Mental Health, and Sensory Organs) Department, Faculty of Medicine and Psychology, Sapienza University of Rome, 00185 Rome, Italy; 4 Department of Mental Health Protection and Promotion, Unit of Addiction Pathology, ASL Rieti, 02100 Rieti, Italy; 5 Bipolar and Depressive Disorders Unit, Hospìtal Clinic de Barcelona. c. Villarroel, 170, 08036 Barcelona, Spain; 6 Departament de Medicina, Facultat de Medicina i Ciències de la Salut, Institut de Neurociències, Universitat de Barcelona (UB), c. Casanova, 143, 08036 Barcelona, Spain; 7 Institut d’Investigacions Biomèdiques August Pi i Sunyer (IDIBAPS), c. Villarroel, 170, 08036 Barcelona, Spain

**Keywords:** treatment-resistant schizophrenia, antipsychotics, dopamine supersensitivity psychosis, clozapine, glutamate, postsynaptic density

## Abstract

Treatment-resistant schizophrenia (TRS) affects up to one in three individuals with schizophrenia and is associated with a significant clinical, social, and economic burden. Different from treatment-responsive forms, TRS appears to involve other biological mechanisms extending beyond dopaminergic dysfunctions. This review outlines current knowledge on the molecular and cellular basis of TRS, focusing on alterations in glutamate signaling, imbalances between excitatory and inhibitory activity, disruptions in D-amino acid metabolism, and evidence of neuroinflammation, oxidative stress, and mitochondrial or endoplasmic reticulum dysfunction. Data from genomics, proteomics, metabolomics, preclinical models, and postmortem studies suggest that TRS may have a peculiar neurobiological substrate. Further, multimodal brain imaging studies reveal differences in brain structure, white matter integrity, and network connectivity when compared to treatment-responsive individuals. Altogether, these findings support a shift from the traditional dopamine hypothesis toward a more comprehensive model that includes multiple immune, metabolic, and synaptic factors. Understanding the possible interplay of these complex mechanisms may lead to the identification of potential biomarkers that may help to predict antipsychotic response, as well as the development of more targeted treatments. Early recognition and a deeper biological insight into TRS are essential for improving care and guiding personalized therapeutic strategies.

## 1. Introduction

Schizophrenia is a severe psychiatric disorder associated with significant personal, social, and economic burden. It compromises quality of life and life expectancy, with individuals affected by the condition experiencing elevated rates of physical comorbidities, unemployment, and social dysfunction [1,2,3]. The illness typically follows a chronic or relapsing course in over 80% of cases [4], and early diagnosis followed by sustained pharmacological treatment has been shown to reduce symptom severity, prevent relapses, and support better functional outcomes [5]. However, many patients do not achieve remission despite optimal treatment conditions, raising critical questions about the underlying mechanisms of treatment resistance.

Treatment-Resistant Schizophrenia (TRS) represents a severe and debilitating form of schizophrenia characterized by a lack of satisfactory response (i.e., less than 20% reduction in the Positive and Negative Symptoms Scale—PANSS) to at least two different antipsychotic trials at adequate dose and for the duration of at least six weeks, according to treatment response and resistance in psychosis (TRRIP) criteria working group [6]. Clinically, TRS is often defined by the persistence of prominent positive symptoms such as delusions and hallucinations, alongside negative symptoms like affective flattening, alogia, and avolition, despite adherence to treatment protocols. TRS is a condition of high epidemiological impact affecting approximately 20–30% of individuals diagnosed with schizophrenia and is responsible for a significant clinical and economic burden [7,8]. While 20–25% of individuals with first-episode schizophrenia exhibit resistance from the onset (primary TRS), an additional 10–20% may develop it over time after an initial response (secondary TRS or dopamine supersensitivity psychosis or DSP) [9,10].

The chronicity and severity of symptoms in TRS contribute to greater functional impairment, higher rates of hospitalization, and poorer quality of life compared to responsive individuals [11]. TRS is also commonly associated with an earlier age of onset, more severe cognitive dysfunction, and distinct neurobiological alterations, indicating a potentially divergent pathophysiology from responsive forms of schizophrenia [12]. The standard of care for TRS is clozapine, the only antipsychotic with proven efficacy and specific indication for this condition [13]. However, its use is frequently delayed due to concerns over adverse effects and the complexity of required monitoring protocols [14]. Such delays are particularly detrimental, as earlier clozapine initiation is associated with improved outcomes [15,16].

Moreover, up to 40–60% of patients with TRS fail to respond adequately even to clozapine, despite optimal dosing and adherence, and are indicated as ultra-TRS (UTRS). These individuals represent a particularly severe and refractory subgroup, highlighting the need for novel therapeutic strategies. Other pharmacological approaches—such as augmentation with mood stabilizers, antidepressants, or electroconvulsive therapy (ECT)—often yield only modest or inconsistent benefits. Given these limitations, a better understanding of the neurobiological mechanisms underlying treatment resistance is a first step to the development of more effective and personalized interventions.

**Figure 1:**
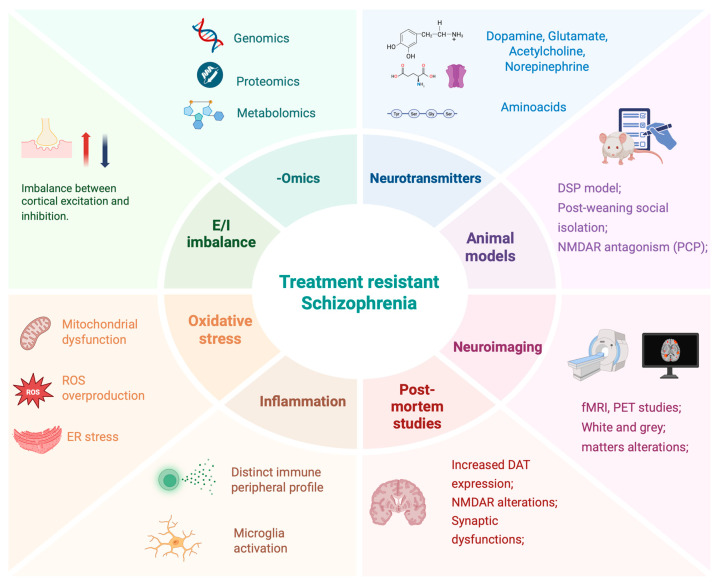
Current biological in vivo and in vitro evidence possibly underlying treatment-resistant schizophrenia. The figure summarizes converging evidence across multiple research levels. Neurotransmitter systems: different neurotransmitter pathways could be implicated in TRS conditions beyond dopaminergic hyperactivity. Both alterations in NE pathways and Ach dysfunctions have been reported, potentially contributing to attention and cognitive disturbances. Moreover, glutamatergic dysfunctions and reduced GABAergic inhibition contribute to altered brain connectivity. Animal models: although there are currently no animal models that can fully represent TRS conditions, several paradigms—including dopamine supersensitivity psychosis (DSP) treatment, social isolation, and NMDAR antagonism—can reproduce features associated with antipsychotic resistance. Neuroimaging: structural and functional studies show cortical thinning, ACC glutamatergic dysfunction, impaired connectivity, and striatal dopamine synthesis dysregulation. Postmortem studies: Studies in TRS patients have shown cortical thinning and neuropil loss, reduced dendritic spine density, altered GABAergic interneurons, and synaptic pathology, as well as evidence of glial activation and redox imbalance. Inflammation: elevated IL-6, TNF-α, IFNγ, and activated monocytes reflect chronic immune deregulation, further supported by evidence of overactivated microglia. Oxidative stress: reduced glutathione, mitochondrial damage, and augmented ROS can cause or amplify neuronal vulnerability. E/I imbalance: excessive glutamatergic drive and reduced GABAergic transmission led to network dysconnectivity. This disruption impairs gamma oscillations, reduces network synchrony, and compromises information processing and cortical connectivity. Genetics and genomics: TRS is enriched in rare disruptive CNVs and elevated PRS, especially within the glutamatergic/GABAergic pathways. Metabolomic and proteomic signatures confirm dysregulated amino acid metabolism and synaptic function. ROS, reactive oxidative species; ER, endoplasmic reticulum; DSP, dopamine supersensitivity psychosis; DAT, Dopamine Transporter; fMRI, functional magnetic resonance imaging; PET, Positron emission tomography; NMDAR, N-methyl-D-aspartate receptor. Created with Biorender.com.

The primary objective of this narrative hypothesis review is to explore the molecular and cellular mechanisms that may underlie the different clinical forms of TRS, with a particular focus on dopaminergic and glutamatergic signaling abnormalities, neuroinflammation, synaptic dysfunction, and genetic vulnerability (Figure 1). By integrating findings from neuroimaging, postmortem studies, and genomic research, we aim to delineate the biological substrates that differentiate TRS from treatment-responsive schizophrenia. In weighing the available evidence, greater weight was given to in vivo studies with larger sample sizes and to those less affected by potential confounders such as chronicity and cumulative antipsychotic exposure. Moreover, we will discuss how these mechanistic insights can inform novel pharmacological targets and guide the development of next-generation therapies. Understanding the molecular landscape of TRS is not only essential for advancing clinical outcomes but also for fostering a more nuanced conceptualization of schizophrenia as a heterogeneous disorder with distinct biological trajectories.

## 2. Putative Neurotransmitter Basis of TRS

### 2.1. Dopamine and Glutamate Hypotheses in TRS

For decades, the dopamine hypothesis has shaped our understanding of schizophrenia and guided its treatment. According to this view, an excess of dopamine activity in the striatum drives the occurrence of psychotic symptoms, a theory supported by imaging studies and the effectiveness of antipsychotics that occupy dopamine D2 receptors (D2R) [17]. Evidence from raclopride positron emission tomography (PET) imaging studies indicates that clinical response to antipsychotics is achieved when a substantial striatal D2R occupancy is reached (60–80%) [18]. Since hyperprolactinemia generally appears after ~73% striatal D2R occupancy [19], and TRS patients often show lower prolactin levels in blood [20], it has been argued that antipsychotics exert an ineffective dopamine blockade in TRS, for instance due to suboptimal or variable D2R engagement, receptor anomalies, or pharmacokinetic differences. However, this explanation does not hold for all patients, raising the question of whether a different biological mechanism might be at play [21].

In secondary forms of TRS, which occurs after an initial period of antipsychotic responsiveness, resistance may arise as a complication of long-term antipsychotic exposure, due to chronic D2R over-blockade, which induces a compensatory upregulation of striatal D2R at postsynaptic sites [22,23]. D2R upregulation may be accompanied by a shift toward a high-affinity receptor conformation, rendering receptors hypersensitive in conditions of endogenous dopamine deficiency [24]. Another dopaminergic mechanism proposed in secondary TRS is the loss of “negative cooperativity” [25,26] among D2Rs, a regulatory process whereby normally dopamine binding to one receptor shifts neighboring receptors to lower affinity states, a regulation that is lost in DSP. Such adaptations increase the vulnerability to rapid relapse upon dose reduction or discontinuation, tolerance to antipsychotics, and often tardive dyskinesia. These forms may respond to clozapine, which, having a weak binding to D2R, reduces compensatory upregulation over time.

Of interest, the use of antipsychotics with predominant dopamine D3 receptor (D3R) antagonism, which are less prone to induce sensitization, or compounds that minimize postsynaptic D2R blockade by acting preferentially at other sites or at a presynaptic level, has recently been explored as a strategy to treat schizophrenia while preventing secondary resistance by avoiding postsynaptic D2R upregulation [27,28,29].

Other regulatory mechanisms at presynaptic sites may be involved in primary TRS; growing evidence indicates that in primary TRS, unlike in responsive patients, presynaptic dopamine synthesis is often not elevated, and a normal presynaptic synthesis capacity is exhibited, highlighting a fundamental difference in dopaminergic regulation [30]. As we know, various studies in the past decades have indicated that increased presynaptic dopamine synthesis may account for a good antipsychotic response [30]. These findings have shifted the focus from postsynaptic dopamine mechanisms to presynaptic mechanisms to better understand TRS neurobiology.

These observations have also spurred interest in neurotransmitter systems other than dopamine, particularly glutamate [31]. The glutamatergic system, and especially the N-methyl-D-aspartate receptor (NMDAR), has emerged as a promising candidate for explaining primary forms of TRS, the so-called ‘normodopaminergic’ subtype. In light of this, many studies focused on altered concentrations in glutamatergic metabolites, particularly using neuroimaging techniques. A recent meta-analysis [32] comparing studies employing proton magnetic resonance spectroscopy (^1^H-MRS), a neuroimaging tool with metabolomic application, reported higher glutamine levels in the putamen of TRS than in individuals responsive to treatment. On the other hand, glutamine levels were higher in the dorsolateral prefrontal cortex of schizophrenia patients than in TRS [33]. In two other small-scale studies, TRS patients exhibited higher glutamate+glutamine (Glx) levels in the dorsal anterior cingulate cortex (ACC) compared to non-TRS [34], and healthy controls [35]. Consistently, increased glutamate concentrations have also been observed in this region [36]. These findings point toward possible disruptions in glutamatergic signaling that may contribute to persistent symptoms independently of dopaminergic transmission; however, they derive from small and methodologically heterogeneous studies, with further longitudinal, prospective studies needed to confirm higher glutamate metabolite levels in the ACC and clarify whether this represents a trait abnormality in TRS.

Importantly, the glutamate hypothesis does not discard the historical dopaminergic model but rather extends it and accounts for other symptom domains (e.g., cognitive and negative ones). It is known that dopamine release in the striatum is regulated, among other mechanisms, by NMDAR modulation [37]. For instance, ketamine pre-treatment in humans has been found to amplify the dopamine release elicited by amphetamines by more than twofold, as measured by single photon emission computed tomography (SPECT) imaging with [^123^I]IBZM, a tracer competing with dopamine for receptor binding. Since ketamine acts as an NMDAR antagonist, affecting corticolimbic γ-aminobutyric acid (GABA)ergic parvalbumin+ (PV^+^) interneurons’ ability to control downstream midbrain dopaminergic neurons [38], this experiment provided evidence for a glutamate–dopamine interaction model in schizophrenia. In this framework, the exaggerated dopamine release may result from NMDAR-mediated prefrontal dysfunction failing to properly regulate subcortical dopamine systems [37].

Thus, dopamine hyperactivity in the mesolimbic pathway—due to either increased release or reduced reuptake—may account for responsive forms of the disease, whereas antipsychotic-induced postsynaptic dopaminergic adaptations may underlie acquired/secondary TRS. On the other hand, glutamatergic dysfunction may act upstream of dopaminergic abnormalities, helping to explain why dopamine-blocking antipsychotics are ineffective in primary TRS; in such cases, glutamatergic dysfunction might be upstream of secondary dopaminergic abnormalities, leading to a failure in the top-down regulation of midbrain dopaminergic activity.

It has been hypothesized that clozapine may exert specific intrinsic or indirect effects on NMDARs—either at the glycine binding site or by inhibiting glycine transporter 1 (GlyT1)—and that its therapeutic target may involve persistently elevated striatal glutamate levels [13]. Within this framework, UTRS may represent the extreme end of the spectrum, in which multiple alterations in dopaminergic, glutamatergic, and other neurotransmitter systems converge, or alternatively, in which the underlying neurobiology remains largely unknown, as suggested by the inefficacy of clozapine.

### 2.2. D-Amino Acid and Glycine Dynamics

D-serine is an amino acid that acts as an endogenous co-agonist at the glycine modulatory site on the GluN1 subunit of NMDAR [39], enhancing its function. Since TRS subjects may present more profound glutamatergic alterations involving NMDAR hypofunction in the prefrontal area, the role of D-serine—along with other co-agonists such as glycine—has gained increasing attention [40,41]. Disruptions in the metabolism, availability, or transport of D-amino acids may impair NMDAR signaling, which could help explain the persistent cognitive and negative symptoms in TRS and why many patients do not respond well to standard dopamine-based treatments [42,43,44]. Several reports have shown that the addition of D-serine as an add-on strategy to standard antipsychotic treatment, may improve negative symptoms and cognition in patients with schizophrenia who are non-responsive [45,46,47]. One study evaluated D- and its precursor, L-serine, levels before and after clozapine treatment. Before clozapine treatment, patients exhibited significantly lower D-serine levels and reduced D-serine/L-serine ratio compared to healthy controls, while no difference was found for L-serine levels. Interestingly, following clozapine treatment, the D-serine/L-serine ratio improved [48]. This suggests that the dysregulation of D-serine metabolism may play a role in the neurobiology of TRS, and that its normalization may contribute to clozapine’s unique efficacy.

These findings suggest that D-amino acid dysregulation and subsequent NMDAR-related dysfunction may be particularly relevant for primary, normodopaminergic, TRS, in which patients show poor response to standard D2-blocking antipsychotics, and the causative abnormalities are thought to lie elsewhere. Clozapine may exert its therapeutic effects in part by enhancing glycine/D-serine pathways, although these actions may be insufficient to overcome the complex neurobiology of UTRS.

Accordingly, several D-amino acid-centered strategies, including supplementation with D-serine, D-aspartate, D-alanine, D-cycloserine, glycine, sarcosine, or inhibition of D-amino acid oxidase (DAO), have been investigated as potential approaches to restore NMDAR function. However, NMDAR neurotransmission manipulation exhibits intrinsic limits due to a “ceiling effect” and a U-shaped dose–response to co-agonist levels, with excessive enhancement being ineffective or even detrimental. Such strategies may be more appropriate as alternatives rather than direct augmentations to clozapine therapy.

### 2.3. Excitatory/Inhibitory Imbalance and GABA Alterations in TRS

The concept of excitatory/inhibitory (E/I) imbalance has become central to understanding the neurobiological underpinnings of TRS. In fact, a growing body of evidence implicates deficient cortical inhibition as a hallmark of schizophrenia broadly, along with pronounced glutamatergic dysregulation in TRS specifically. This observation has led investigators to hypothesize that heightened glutamatergic activity, and by extension, increased cortical excitability may contribute more substantially to TRS than dopaminergic dysfunction alone. Transcranial magnetic stimulation (TMS) paradigms, particularly short-interval intracortical inhibition (SICI), have been utilized to probe the functional integrity of inhibitory GABAergic circuits. Meta-analytic data consistently demonstrate a significant reduction in SICI among individuals with schizophrenia, indicating a robust inhibitory deficit that manifests as decreased GABAergic tone [49]. A recent study replicated these findings in TRS patients, showing a significant increase in E/I index and marked SICI impairment also in treatment-refractory individuals [50]. E/I imbalance may arise from a combination of excessive excitatory drive and weakened inhibitory control. As outlined before, aligning with the detection of elevated E/I ratio, several neuroimaging and spectroscopic studies report localized increases in cortical glutamate concentrations among TRS patients, notably within the cingulate cortex [32,36,51], further supporting the notion of a shift toward cortical hyperexcitability in TRS.

Since SICI reflects a specific GABA-A receptor-mediated inhibition [52], while LICI is thought to reflect GABA-B receptor activity [53] and appears preserved in TRS, a progressive and selective dysfunction in GABA-A inhibitory pathways may shape the cortical disinhibition characteristic of TRS. Notably, individuals with non-TRS exhibit intermediate SICI deficits, pointing to a continuum of GABA-A dysfunction severity across the schizophrenia spectrum. Moreover, the same report indicated a significant positive correlation between SICI and negative and autistic symptoms, suggesting that impaired GABA-A inhibition may drive symptoms resistant to dopamine antagonists such as avolition, social withdrawal, and cognitive deficits that often characterize TRS and overlap with autistic features [50]. However, it should be noted that these results derive from a single study, whose design does not stratify patients into primary, secondary, and UTRS subgroups due to the small sample size, and in which all subjects are exposed to antipsychotics and TRS exposed to clozapine. Clozapine is known to increase extracellular glutamate and modulate GABA release in specific brain regions [13] and, thanks to its unique pharmacodynamic profile, it may be able to reduce cortical inhibition deficits better than other antipsychotics, attenuating potential differences between TRS and non-TRS patients. In brief, this shift toward excitation has been reported not only in patients with schizophrenia, as confirmed by a recent meta-analysis of TMS–electromyography (EMG) paradigms, but also in a small unstratified sample of TRS subjects and in experimental models of secondary TRS, where DSP is induced through specific protocols.

### 2.4. Cholinergic Modulation of Dopaminergic and Glutamatergic Circuits in Treatment-Resistant Schizophrenia: From Nicotine to Muscarinic Agonists

Dopaminergic, GABAergic, and glutamatergic signaling can all be significantly modulated by the activation of the cholinergic system [54], through nicotinic acetylcholine receptors (nAChRs) and muscarinic acetylcholine receptors (mAChRs), which have been shown to influence neural circuitry disrupted in schizophrenia [55]. These receptor families are differentially implicated in symptom domains, with nAChRs primarily associated with cognitive enhancement and mAChRs with broader symptom modulation.

Of interest, people with schizophrenia smoke more often than the general population and tend to be heavier smokers [56,57,58,59,60,61,62] for this reason, alterations in nicotinic neurotransmission have been suspected in psychotic patients. Moreover, the typical pro-cognitive effects of nicotine observed in the general schizophrenia population may not be evident in TRS [63,64]. Tobacco smoking may fail to enhance, or may even worsen, prefrontal dopaminergic transmission, exacerbating the underlying hypodopaminergia linked to cognitive deficits and negative symptoms. A dysfunctional interplay between nicotinic and glutamatergic systems, both implicated in TRS pathophysiology, could contribute to the observed worsening in cognitive performance and clinical symptoms among smoking TRS patients [65].

A peculiar subtype of nicotinic receptor is the α7 nAChRs, which has increasingly been implicated in several neuropsychiatric disorders, including schizophrenia, drug addiction, depression, anxiety, and multiple neurodegenerative disorders [66,67,68,69,70,71,72].

Single-nucleotide polymorphisms (SNPs) of the promoter region of α7 nAChRs gene (*CHRNA7*) are more frequent in people with schizophrenia [73]. Furthermore, this receptor has been associated with impaired auditory gating, namely an altered P50 suppression, index of pre-attentive sensory gating measured with a paired-click paradigm. Healthy subjects show a reduced P50 response to the second click because early inhibitory circuits (notably, α7-nAChR–dependent hippocampal interneurons) are engaged. In schizophrenia, this attenuation is reduced, indicating deficient sensory gating [74].

A recent review and meta-analysis by Saint-Georges et al. [75] has synthesized evidence from in vivo neuroimaging studies demonstrating significant regional decreases in cholinergic receptor availabilities. In particular, three positron emission tomography (PET) studies using [18F]ASEM, an α7 nAChR antagonist, showed reduced binding in cingulate, frontal, parietal, temporal, occipital cortices as well as in subcortical regions including thalamus, striatum, and hippocampus [76,77,78] of schizophrenia patients compared to healthy individuals. A recent dual tracer PET study assessed both α7 nAChR availability and glial activation by mapping the 18 kDa translocator protein (TSPO) levels performing two same-day scans in 19 patients and 20 healthy individuals [79]. The authors found positive correlations between TSPO levels, α7 nAChR availability and cognitive performance, in particular letter fluency, across multiple brain regions, suggesting an interplay between the cholinergic signaling and the neuroinflammatory processes.

Consistently, also several postmortem investigations have reported decreased α7 receptor expression in brain tissue from individuals with schizophrenia, in particular in the thalamus, hippocampus, frontal, and cingulate cortex [80,81,82].

Preclinical works have further shown that presynaptic α7 nAChRs, together with β2 subtypes, directly regulate glutamate release from frontal cortex terminals in a Ca^2+^-dependent manner, thereby modulating downstream NMDAR activation [83]. Moreover, stimulation of astrocytic α7 nAChRs is able to induce alpha-amino-3-hydroxy-5-methyl-4-isoxazolepropionic acid receptor (AMPAR) recruitment on glutamatergic synapses in hippocampal neurons, thus converting ‘silent’ glutamatergic synapses to functional status [84].

Thus, α7 nAChR dysfunction might contribute to NMDAR hypofunction and plasticity deficits, mechanisms strongly implicated in TRS pathophysiology. Disturbances in this cholinergic–glutamatergic interplay could underlie persistent symptoms in primary TRS, highlighting α7 nAChRs as a potential therapeutic target beyond dopamine blockade. It is noteworthy that clozapine, unlike other antipsychotics, can restore the auditory sensory gating impairment toward near-normal levels, possibly through an α7 nAChR mechanism [85,86].

Altogether, direct evidence for α7 nAChR involvement in TRS is still lacking; yet converging PET, postmortem, and mechanistic data render α7 dysregulation biologically plausible for sensory gating and cognitive/negative domains. We have to acknowledge clinical neuroimaging studies’ limitations: PET investigations are often underpowered, sample sizes are constrained by radioactive tracer exposure, confounding from smoking status, and heterogeneity in illness duration; similarly postmortem studies are limited by selection and agonal factors, medication and substance-use histories, and most importantly, uncertainties in diagnostic reliability.

We therefore posit that α7 dysfunction is unlikely to be a unitary driver of TRS but may act as a disease-modifying contributor in a percentage of subjects. Since orthosteric α7-nAChR agonists have shown disappointing clinical efficacy and failed to translate, current development is shifting toward positive allosteric modulators (PAMs) and ago-PAMs.

Regarding mAChRs, there are five subtypes of metabotropic muscarinic receptors (M1-M5): M1, M3, and M5 typically couple with G_αq_ protein, activating phospholipase C, leading to inositol trisphosphate production, and intracellular calcium (Ca^2+^) release, whereas M2 and M4 couple with G_αi_ proteins, inhibiting adenylyl cyclase activity and reducing cAMP levels [87]. Increasing evidence supports the involvement of mAChRs, particularly for M1, M4, and M5, as potential therapeutic targets [54]. This is further supported by findings that muscarinic antagonists can induce schizophrenia-like symptoms in healthy individuals [88] and exacerbate positive symptoms in patients [89].

For decades, mAChRs have been considered potential pharmacological targets for the treatment of schizophrenia, particularly as cognitive enhancers. This interest is supported by growing evidence from both clinical and preclinical studies demonstrating robust pro-cognitive effects of compounds that directly or indirectly activate mAChRs. However, a major limitation to their clinical application is the low subtype specificity of these compounds, which often leads to classical peripheral cholinergic side effects. In fact, the acetylcholine binding site at mAChRs, is highly conserved across subtypes, making it challenging to design pharmacological agents with meaningful subtype selectivity [54]. Xanomeline, one of the most extensively studied mAChR agonists, has been tested in two separate clinical trials involving patients with Alzheimer’s disease and schizophrenia. In both cases, xanomeline was associated with improvements in clinical symptoms [90,91,92]. However, its development was initially hampered by significant gastrointestinal adverse effects, which limited its consideration in routine clinical practice. This limitation was subsequently addressed by combining xanomeline with trospium, a non-selective mAChR antagonist that, due to its inability to cross the blood–brain barrier (BBB), selectively targets peripheral receptors, thereby mitigating the side effects associated with peripheral cholinergic activation [93,94,95]. This pharmacological association was evaluated in a clinical trial involving patients with schizophrenia, demonstrating good tolerability and significant reductions in PANSS scores. On 26 September 2024, the xanomeline–trospium combination has received approval from the United States (US) Food and Drug Administration (FDA) for the treatment of schizophrenia, based on data from two 5-week, randomized, double-blind, placebo-controlled, multicenter studies involving 470 adults with schizophrenia [96,97].

No evidence is currently available to support the use of muscarinic antagonists in the treatment of TRS, despite growing interest in this field, as demonstrated by recent studies involving the M4 positive allosteric modulator emraclidine in patients with schizophrenia, though not specifically in TRS [98].

By contrast, it is well established that N-desmethylclozapine (norclozapine), the active metabolite of clozapine, acts as an agonist or positive allosteric modulator at M1 and M4 receptors, enhancing central cholinergic and glutamatergic transmission. This receptor profile is thought to contribute to clozapine’s unique clinical efficacy in TRS, particularly in addressing cognitive deficits. Given that norclozapine’s M1/M4 agonist activity may drive these effects—potentially via downstream glutamatergic pathway modulation—there is a compelling rationale to explore whether other agents with similar mechanisms could yield therapeutic benefits in primary TRS. In this context, xanomeline, a direct M1/M4 receptor agonist, warrants further investigation as a potential core or adjunctive treatment strategy for TRS [99] (de Bartolomeis, 2022).

### 2.5. The Noradrenergic System in Treatment-Resistant Schizophrenia: A Neglected Neurotransmitter Pathway?

The noradrenergic hypothesis of schizophrenia proposes that alterations in norepinephrine transmission are responsible for the development of psychotic symptoms, partly due to the interaction of the noradrenergic system with dopamine and glutamate [100,101].

Norepinephrine is mainly contained in the locus coeruleus. Connections branch out from this structure to the prefrontal cortex, amygdala, thalamus, basal ganglia, and hippocampus, thus playing an essential role in functions such as memory, sustained attention, arousal, and stress response [102,103,104,105,106]. A dysfunction in the noradrenergic system could therefore alter these functions, leading to psychotic symptoms, cognitive alterations, and attention deficits, which are characteristic of patients with schizophrenia [101].

It has been suggested that increased noradrenergic activity is responsible for positive symptoms (delusions and hallucinations) [107], and that a decrease in norepinephrine is correlated with negative symptoms (anhedonia, anergy, abulia, social withdrawal). Thus, the noradrenergic system may influence multiple symptom domains for schizophrenia. Several clinical studies have demonstrated a correlation between acute psychotic symptoms, mainly paranoid symptoms, and elevated levels of norepinephrine in the serum and cerebrospinal fluid (CSF) of patients [108]. Plasma levels of norepinephrine metabolite, 3-methoxy-4-hydroxyphenylglycol (MHPG), have been correlated with greater severity of negative symptoms in drug-free schizophrenia subjects by Pickar and colleagues [109]. These findings are limited and not entirely consistent with the classical hypothesis. Moreover, MHPG measured in plasma reflects both central and peripheral norepinephrine metabolism, whereas CSF metabolites more directly index central noradrenergic activity. Thus, this apparent discrepancy may reflect complex noradrenergic tone regulation, differential effects across central vs. peripheral compartments, or compensatory mechanisms. These aspects warrant further investigation. 

In TRS patients, where the typical antipsychotic treatments do not give an adequate response, other neurotransmitter pathways, like the noradrenergic system, should be examined to understand their role further. 

Since stress-related outbursts of noradrenaline from the locus coeruleus may potentiate the dopaminergic burst firing via alpha-1 (α 1) adrenergic receptor in ventral tegmental area (VTA) and striatal circuits, weakening top-down prefrontal control; under conditions of dopamine sensitization, it is plausible that also modest norepinephrine elevations amplify phasic dopamine release, lowering the threshold for psychotic breakthrough despite D2R blockade. Thus, these mechanisms are more consistent with secondary TRS, DSP condition, than with primary TRS.

In brief, while norepinephrine is critical in brain processes related to stress, attention, and arousal applicable to schizophrenia, its precise role and therapeutic targeting in TRS remain an area for further research rather than established clinical knowledge.

## 3. Insights from “Omics” Studies (Genomics, Proteomics, Metabolomics) in TRS

### 3.1. Genomics Studies on TRS

Schizophrenia is considered a disorder with high heritability, with a mean from recent twin studies of 81% [110]. The Schizophrenia Working Group of the Psychiatric Genomics Consortium identified 104 loci associated with an increased risk of schizophrenia [111]. A more recent update expanded this to 120 loci, 104 of which encode proteins that contribute to the risk of developing the disorder [112]. It has been suggested that non-responsive schizophrenia could represent a different phenotype underlying specific genetic alterations different from schizophrenia responsive to treatment [8]. Nevertheless, few studies have investigated specific genetic differences in TRS patients. Candidate genes studies focused on the dopaminergic, serotoninergic, glutamatergic and GABA systems as well as inflammatory and oxidative systems.

Only a few large-scale studies have focused specifically on TRS. A genome-wide association study (GWAS) of two Caucasian cohorts, compared patients with TRS (n = 79 and n = 70) to without TRS (n = 95 and n = 125) [113]. No variant reached genome-wide significance (*p* < 5 × 10^−8^), but a suggestive signal emerged at rs2237457 on 7p12 (combined *p* = 5.66 × 10^−7^), located upstream of aromatic L-amino acid decarboxylase (DDC), an enzyme critical for monoamine synthesis—e.g., conversion of 3,4-dihydroxyphenylalanine (L-DOPA) to dopamine and 5-HTP to serotonin. Further, polymorphisms of the *type three metabotropic glutamate receptor 3* gene (*GRM3*) have been related to refractory psychotic symptoms [114]. Metabotropic glutamate receptor 3 (mGluR3), encoded by the *GRM3* gene, modulates glutamate neurotransmission through NMDAR, which are implicated in cognition and negative symptoms in schizophrenia [115]. Moreover, some variants of both *glutamate decarboxylase 1* (*GAD1*) and *GABA-B receptor subunit 2* (*GABBR2*) genes were weakly related to treatment-resistant patients [116], altering the GABA neurotransmission and the intracellular transduction signaling, respectively. A few studies associated a specific dopamine D1 receptor gene (*DRD1*) polymorphism (rs4532) with TRS [117,118,119]. Other polymorphisms associated with treatment resistance are *OXT* rs2740210 C-allele and *OXT receptor* rs2228485 A-allele, which involve the oxytocin and oxytocin receptor genes, respectively [117]. Oxytocin is involved in blunted affect and social withdrawal, which are core features of the negative symptom domain; thus, variations in these loci may contribute to refractoriness to antipsychotic treatment, particularly in patients with predominant negative symptoms.

Additionally, variants in the brain-derived neurotrophic factor (*BDNF*) gene have been proposed as potential contributors to TRS [120,121]. For instance, variants including the Val66Met (rs6265) polymorphism have been implicated in TRS [120,121]. The Met allele has been associated with reduced activity-dependent BDNF secretion and subsequent impaired glutamatergic and dopaminergic signaling, and altered synaptic plasticity, potentially limiting the neuroadaptive mechanisms underlying antipsychotic efficacy.

Other variants are not explicitly associated with TRS in large GWASs but related to poor response have been highlighted in the literature. Specifically, greater evidence was found for the rs1799732 (−141C Ins/Del) and rs1800497 dopamine D2 receptor gene (*DRD2*) polymorphisms. The rs1799732 is an SNP located in the promoter region of the gene. It appears to be associated not with schizophrenia risk [122] but specifically with the treatment response, as it affects gene expression [123,124]. A study by Lencz T. et al. [125] showed that −141C Ins/Del carriers on DRD2 genes needed a longer time to respond to treatment, suggesting that specific polymorphism on dopamine receptor-related genes may have a role in the treatment response.

Common variants of genes involved in serotonin transmission may influence the treatment response, as serotonin receptors are the targets of many antipsychotic drugs. Specifically, the polymorphism rs6313 (T102C) of the *HTR2A* gene (encoding for 5-hydroxytryptamine receptor 2A) is more common in non-responsive patients [126], as are several *HTR2C* (encoding for 5-hydroxytryptamine receptor 2C) polymorphisms [127]. Additionally, one study found that the His452Tyr (H452Y, rs6314) variant in the *HTR2A* gene was associated with a lack of response to clozapine [128]. Both serotonin transporter and receptor genes are implicated. A polymorphism in the promoter region of the *SLC6A4* gene (encoding for the serotonin transporter—SERT), serotonin-transporter-linked promoter region (5-HTTLPR, short/long alleles), is associated with reduced SERT expression [129] and reduced response to antipsychotics [130,131]. Moreover, individuals homozygous for the short form were more likely to be treatment-resistant [132].

In recent years, dysfunctions of glutamate neurotransmission have been reported as a possible mechanism [9,133] of TRS, as well as the involvement of GABA-altered transmission [134].

Genetic variability in drug-metabolizing enzymes may affect treatment response in schizophrenia. For instance, variants in *cytochrome P450* genes, particularly *Cytochrome P450 (CYP)2D6*, *CYP1A2*, *CYP3A4*, and *CYP2C9*, which are key in the metabolism of many antipsychotics, have been associated with poor clinical outcomes [135,136,137,138,139]. Subjects with variants that make CYP activity very efficient (rapid or ultrarapid metabolizers) may exhibit lower plasma concentrations of the drug at standard doses, resulting in subtherapeutic exposure. This can be misinterpreted as treatment resistance, while in fact representing a pharmacokinetic issue that should be considered a form of pseudo-resistance. On the other hand, “poor metabolizers” carrying variants that reduce cytochrome activity may experience intolerable side effects due to elevated plasma levels. Intolerability or drug discontinuation due to adverse events can mimic resistance; thus, the patient may be labeled as TRS, while the real issue is toxicity or non-adherence. In this framework, the TRRIP guidelines emphasize the importance of ruling out pseudo-resistance before diagnosing TRS [6].

Moreover, several catechol-O-methyltransferase (COMT) variants have been investigated concerning TRS status since COMT metabolizes catecholamines and regulates dopamine degradation [140,141]. In particular, the Val158Met (rs480) polymorphism in the *COMT* gene results in at least a fourfold reduction in enzyme activity, leading to increased dopamine availability [142], which may contribute to reduced response to antipsychotic treatment.

Similarly, polymorphisms in drug transporters also play an important role: *SLC22A1* (encoding the organic cation transporter 1—OCT) regulates hepatic drug uptake, and defective alleles (*3, *5*) reduce metabolism and increase systemic exposure; *ABCB1* (encoding P-glycoprotein, a multidrug resistance transporter) variants, such as C1236T, C3435T, and G2677A/T, influence intestinal absorption and BBB penetration, thereby altering efficacy and side-effect burden [143,144]; ATP-binding cassette subfamily C member 2 protein (*ABCC2*), also known as multidrug resistance-associated protein 2 (MRP2), polymorphisms, such as rs717620, have similarly been linked to reduced efflux and greater olanzapine exposure [137]. Variants in Apolipoprotein C-III (*APOC3*), a gene involved in triglyceride transport, may modify antipsychotic distribution due to its lipophilic nature, contributing to altered pharmacokinetics [137].

One study revealed a significant association between TRS and the overall burden of genome-wide copy number duplications [145]. This may suggest that rare genetic variants, such as copy number variations (CNVs), may play a more prominent role in the genetic architecture of TRS compared to common single-nucleotide variants captured by polygenic risk score (PRS). Individuals affected by severe and treatment-resistant forms of schizophrenia appear to carry a greater burden of rare damaging variants in genes intolerant to variation compared to controls [146]. These rare variants often disrupt genes involved in synaptic function and neurodevelopment [147], including recurrent duplications of 15q11.2-q13.1 and deletions of 16q11.2, which have been implicated in TRS pathophysiology.

Finally, in recent years, TRS genetic studies have focused on haplotypes, namely a combination of genetic variants (usually SNPs) inherited together because of their physical proximity on a chromosome, for instance, within the *DRD2* and *HTR2A* loci, yielding contrasting results [123,128]. Dedicated, well-powered haplotype-based studies in TRS are still sparse, and further research is required to validate this approach.

In summary, while candidate gene studies have highlighted variants in dopaminergic, serotoninergic, and glutamatergic pathways, GWAS and PRS analyses point to a complex polygenic and heterogeneous architecture of the disease, with rare variants likely playing a crucial role in driving TRS.

Despite significant progress in understanding the genetic underpinning of TRS, current findings remain contrasting and heterogeneous for several reasons. First, the definition of TRS varies across studies. Second, in GWAS, different significance thresholds have been applied when calculating PRS. Third, many studies lack sufficiently large sample sizes, and often do not include populations from different ancestries, which would be essential to improve the robustness of the findings. Additionally, most PRS studies focus on loci associated with general schizophrenia risk, but it would be valuable to also consider loci specifically related to treatment response, early age at onset and poor prognosis.

### 3.2. Proteomics and Metabolomics

Proteomic studies in schizophrenia have analyzed samples from various tissue sources, including postmortem brain tissue, CSF, serum, and plasma [148]. These investigations have highlighted several biological alterations, such as the downregulation of postsynaptic density proteins [149], disruption in energy metabolism, mitochondrial dysfunction, oxidative stress [150,151], and dysregulation of immune-inflammatory response. Despite the growing body of research, proteomic studies specifically focusing on TRS are limited, with most of the studies exploring systems already implicated in schizophrenia, such as the immune-inflammatory system. In parallel, metabolomic studies offer insight into the biochemical alterations underlying TRS patients. By analyzing metabolic profiles, this field provides novel information for understanding disease mechanisms and possible biomarkers of treatment response. Among the first applications of these studies have been the metabolic alterations linked to neurotransmitter dysregulation.

Two independent proteome-wide association studies (PWAS) conducted on GWAS data comparing TRS and non-TRS (10,501 TRS vs. 20,325 non-TRS) pointed 41 proteins differentially expressed, two of which were duplicated in the PWAS analysis of the discovery and confirmation datasets, suggesting a potential role in TRS, including carnitine palmitoyl transferases 2 (CPT2) and apolipoprotein L2 (ApoL2) [152]. ApoL2 is indirectly linked to the inflammatory processes, as it mediates interferon-gamma (IFN-γ)-induced cell death [153], suggesting a link between immune dysregulation and resistance to antipsychotics. CPT2 is a component of a multiprotein catalytic complex embedded in the mitochondrial membrane, where it enables long-chain fatty acids to enter the mitochondrial matrix for β-oxidation and energy production. Specific loss of CPT2 may lead to altered lipid-oxidation processes and contribute to mechanisms underlying TRS. Oxidative stress appears to be a key pathway implicated in TRS. PWAS pointed also to peroxiredoxin 1 (PRDX1), a protein involved in reducing oxidative stress by neutralizing ROS [154,155] as significantly associated with TRS [152]. Antipsychotic drugs have been shown in vitro to influence PRDX1 expression. Also, disruptions in the trans-sulfuration pathway may reduce selenoprotein synthesis, contributing to increased oxidative stress in TRS [156].

Another potential pathway implicated in TRS is the kynurenine pathway (KP). Kynurenic acid (KYNA) and quinolinic acid (QUIN) are the principal metabolites of the KP of tryptophan degradation. KYNA is an allosteric antagonist of the NMDAR, promoting channel closure, and has been implicated in schizophrenia pathogenesis [157]. Conversely, QUIN acts as an agonist at the glutamate orthosteric site of NMDARs [158]. If a meta-analysis suggested increased KYNA levels in schizophrenia patients, specifically within the central nervous system (CNS), the evidence regarding the KP in TRS patients remains inconsistent. Some studies found lower KYNA plasma levels in non-TRS [159,160], while other authors reported higher saliva KYNA levels in TRS than in non-TRS [161]. In contrast, other reports did not find differences in KP metabolites between TRS and non-TRS [162], and no significant differences for QUIN levels have been reported [161].

Thus, clear and consistently replicated alterations in KYNA and QUIN levels have not yet been demonstrated. But KP is mechanistically attractive because it links immune activation to glutamatergic tone and redox biology [158,163,164,165]. Through these mechanisms, KP abnormalities may reproduce glutamatergic hypofunction, and further targeted investigations in TRS are warranted.

Overall, proteomic and metabolomic data in TRS converge on synaptic/mitochondrial dysfunction, redox stress, and immune–metabolic imbalance, but findings remain heterogeneous across tissues (brain vs. CSF vs. plasma/saliva) and are confounded by medications, smoking, diet, and illness stage.

### 3.3. Animal Models of TRS

Animal models specifically designed for TRS are less numerous and less extensively characterized compared to those for general schizophrenia, but emerging evidence suggests that certain preclinical paradigms may reproduce TRS pathophysiology in some behavioral and biological aspects.

One notable feature that has gained attention is the role of prolonged exposure to antipsychotics, which can lead to DSP. As outlined before, chronic exposure to high-potency D2 receptor-blocking antipsychotics can induce a sensitized dopaminergic state in a subset of animals, due to D2R compensatory upregulation and an increase in their affinity [166,167] leading to exaggerated behavioral responses and poor treatment response. Approximately 40–60% of chronically exposed animals display enhanced locomotor responses and are classified as DSP-positive, while the remaining animals may not develop this condition. For instance, Oda and colleagues (2017) [168] investigated this phenomenon by administering haloperidol chronically to rats (0.75 mg/kg/day for 14 days via osmotic mini-pump), observing that some animals developed DSP, characterized by altered glutamatergic signaling in the brain. Specifically, DSP rats exhibited an increased ratio of GABA to glutamate and changes in related enzymes and receptor subunits in the striatum, such as elevated glutamic acid decarboxylase (GAD) and serine hydroxymethyltransferase (SHMT2), alongside a relative decrease in NMDAR function in non-DSP rats. These findings suggest that the balance between inhibitory and excitatory neurotransmission is shifted in DSP, which may underlie the neurobiological basis of treatment resistance.

Complementing these findings, Tadokoro et al. [169] explored the effects of different antipsychotic treatments on dopamine supersensitivity in rats. Chronic treatment with haloperidol (0.75 mg/kg/day via osmotic mini-pump) but not aripiprazole increased both locomotor responses to stimulants and striatal D2R density, markers of dopamine supersensitivity. Together, these studies provide a robust animal model for understanding secondary TRS centered on dopamine supersensitivity induced by chronic D2R blockade. Nonetheless, animal models specifically addressing primary TRS are scarce.

Beyond supersensitivity models, other preclinical paradigms have also shown relevance for other TRS subtypes, especially for their ability to model features poorly responsive to standard antipsychotics, such as negative symptoms and cognitive deficits.

It should be noted that developing animal models of primary TRS is more challenging compared with DSP. While the biological underpinnings of DSP are relatively well characterized and can be experimentally reproduced, in the case of primary TRS researchers can only mimic the clinical phenotypes without knowing the precise biological lesions to replicate. For this reason, current animal studies provide only limited translational insight into the pathophysiology of primary TRS. The postweaning social isolation model, which induces behaviors resembling the negative symptoms of schizophrenia such as neophobia, social withdrawal, and cognitive deficits [170,171], is also particularly relevant for TRS, since negative symptoms tend to be more severe in treatment-resistant patients. In this model, the hyperactivity can be reduced by blocking D3R but not D2R, reflecting the poor response to typical antipsychotics observed in many TRS cases [172]. Also, models based on NMDAR antagonism, such as chronic phencyclidine (PCP) administration, cause behavioral and cognitive alterations that do not respond to typical antipsychotics but partially improve with clozapine treatment [173]. For instance, it has been described that reduced social behavior in mice treated with PCP for 14 days was improved by clozapine treatment but not by haloperidol [174], mirroring the clinical profile of TRS and highlighting the possible role of glutamatergic dysfunction in its pathogenesis. 

Overall, animal models of TRS emphasize the relevance of dopaminergic supersensitivity, but social isolation and NMDAR antagonist models have also provided insights, especially into negative symptoms and cognitive deficits resistant to D2R-based interventions. Clozapine’s efficacy in these models supports the idea that TRS involves mechanisms beyond dopaminergic dysregulation, possibly including glutamatergic and inhibitory pathways. Patients with TRS differ not only in symptom profiles but also in treatment history, illness duration, cognitive function, and comorbidities; these aspects are rarely reproduced in preclinical settings. Most models focus on isolated mechanisms or symptom clusters, lacking the multidimensionality of real-world clinical presentations. Moreover, the efficacy of antipsychotics is frequently overestimated in animal models, especially because immediate medication effects are prioritized above long-term therapy results. In contrast, clinical treatment involves chronic administration and delayed therapeutic effects, with issues such as tolerance, side effects, and long-term functional outcomes playing a central role. Therefore, because they rely on simplified behavioral measures (such as locomotor activity) that do not fully capture the complexity of human psychopathology and often use equally simplified indicators of treatment response, animal models of TRS have limited translational validity.

### 3.4. Postmortem Evidence of Neurochemical Alterations in TRS

Neurobiological differences between treatment-responsive and treatment-resistant schizophrenia subjects have been rarely studied in postmortem tissue. A study by Purves-Tyson and colleagues examined midbrain tissue, reporting significantly higher dopamine transporter (DAT) protein levels in patients thought to be treatment-resistant, compared to putatively treatment-responsive cases, pointing to a possible biological marker of poor response to antipsychotics [175]. Abnormalities of N-methyl-D-aspartate (NMDA) receptor 1 subunit (NR1) subunits in NMDARs have been linked to schizophrenia in a series of studies, among which postmortem studies can provide direct evidence in the brains of schizophrenia patients.

Research on NMDARs in postmortem brain tissue from individuals with schizophrenia was first reported in 1989 by Kornhuber and colleagues. Using radioligand binding with [^3^H]MK-801, which targets the PCP site of the NMDAR channel, they did not detect any diagnostic differences in the prefrontal cortex or hippocampus compared with controls [176]. Since then, a number of studies have continued to probe the idea of abnormal NMDAR function by examining the binding and gene transcription levels of individual NMDAR subunits in various brain regions.

Another study explored differences in synaptophysin, vesicular glutamate transporter 1 (vGLUT1), calcineurin, and Mitofusin-2 expression as a marker of synaptic activity in the ACC in schizophrenia and TRS. Only synaptophysin and calcineurin were related to schizophrenia, while no difference was found for the other proteins [177]. Synaptophysin is often used as a marker of axon terminals [178], while calcineurin is implicated in regulating synaptic vesicle endocytosis [179], and reduced levels of these proteins in ACC may lead to altered signal transduction, which may contribute to emotional dysregulation and impaired decision-making. 

Regarding oxidative stress, one postmortem study conducted on striatal tissues reported a 40% reduction in mitochondria per synapse in the caudate nucleus and putamen in schizophrenia patients but not in TRS [180], highlighting a biological difference between treatment response and treatment resistance.

In conclusion, there remains a significant gap in postmortem research specifically focused on TRS, as most studies have addressed general schizophrenia pathophysiology. Moreover, postmortem studies face methodological challenges, including retrospective diagnostic labeling, long illness duration, small sample sizes, and chronic antipsychotic exposure. Consequently, these findings should be interpreted with caution and cannot be considered conclusive mechanistic evidence.

## 4. Inflammation and Immune System Involvement in TRS Pathophysiology

Although inflammation and immune dysregulation have long been implicated in schizophrenia, these mechanisms may play a particularly central role in TRS [158]. TRS patients, characterized by marked cognitive deficits, neurobiological alterations, and poor outcomes, often exhibit distinct immune-inflammatory profiles compared to treatment-responsive individuals [181,182]. A growing body of evidence from clinical, preclinical, and postmortem studies supports the hypothesis that immune activation contributes to the pathophysiology of resistance to antipsychotics [183,184,185,186,187]. This line of research connects back to the neurodevelopmental model of schizophrenia, in which early immune challenges, particularly maternal infections, may trigger inflammatory cascades that alter brain maturation [188,189]. These alterations can persist into adulthood, potentially shaping vulnerability to TRS.

Prenatal infections and maternal immune activation (MIA) lead to increased placental and fetal inflammation, microglial activation, and oxidative stress, all of which may interfere with neuronal development [190,191]. In particular, redox imbalance, which has been related to TRS, may exacerbate these neurodevelopmental vulnerabilities [192]. TRS patients often show persistent oxidative stress and impaired antioxidant defenses, such as reduced superoxide dismutase and catalase activity, which may contribute to ongoing neuronal damage [192].

During MIA, pro-inflammatory mediators, such as cytokines (tumor necrosis factor—TNF-α, interleukins (IL)-1β, IL-6), chemokines, antibodies, and acute phase proteins are released, which increase placental and fetal BBB permeability, activate microglial cells, and alter processes fundamental to normal brain maturation [190,193]. Studies revealed that individuals with schizophrenia exhibit multisystem biological dysregulations in peripheral blood, with alterations in circulating immune system proteins and elevated levels of IL-6 in both peripheral blood and CSF, suggesting a potential causal relationship between the IL-6/Interleukin-6 receptor (IL-6R) pathway and schizophrenia [194]. Elevated IL-6 levels have been consistently reported in patients with TRS [195,196,197,198]. Interestingly, IL-6 levels often decrease following antipsychotic treatment, indicating their potential utility as biomarkers of treatment response, particularly in TRS [199].

The inflammatory profile of TRS appears distinct not only in cytokine levels but also in regulatory mechanisms. While non-TRS patients show moderate elevations in IL-6 and its soluble receptor (sIL-6R), TRS patients tend to exhibit more pronounced increases [183]. This heightened inflammatory response is accompanied by reductions in endogenous anti-inflammatory proteins, such as Clara cell protein 16 (CC16), suggesting a putative compensatory imbalance in immune regulation [200]. Supporting this, CC16 has been shown in vitro to modulate IFN-γ signaling and protect against inflammatory reactions [185].

Further evidence, while confirming that schizophrenia patients have higher levels of IL-6 than healthy controls, have documented a significant increase in IL-1 receptor antagonist (IL-1RA)—an endogenous inhibitor of the pro-inflammatory cytokine IL-1 effect- in TRS patients compared with healthy controls and non-TRS patients, together with a specific activation of the monocytic arm of cell-mediated immunity [186,201]. Such an increase may represent a compensatory mechanism or reflect enhanced IL-1 activity driven by a pronounced inflammatory state in TRS. Antipsychotics appear to modulate IL-1 and IL-1RA expression; in particular, clozapine has been shown to induce pro-inflammatory cytokine production during short-term treatment, whereas prolonged exposure is associated with elevated plasma concentrations of IL-1RA [202].

Within this framework, UTRS exhibits even more pronounced immune alterations. Whereas TRS patients respond to clozapine and exhibit elevations in IL-12/IL-23p40, IL-17A, IL-10, and beta-2 microglobulin (B2M), UTRS patients display additional increases in IL-6, IFNγ, and TNF-α, pointing to a more robust activation of the Th17 pathway and associated pro-inflammatory network [196,203,204].

These findings suggest that different immune profiles may significantly influence the response to antipsychotics as well to clozapine treatment, indicating the potential for immune-related indicators to predict the likelihood of symptom improvement before initiating antipsychotic therapy [195].

However, research on immune biomarkers in TRS shows heterogeneous and not always convergent results, exemplified by differences observed in C-reactive protein (CRP) and IL-8 levels. CRP, a well-known marker of systemic inflammation, is often elevated in patients with schizophrenia, and some studies indicate an association with treatment resistance [205]. However, other studies do not confirm this correlation [206,207,208]. Similarly, IL-8 levels show conflicting data, with no clear or consistent evidence regarding their link to TRS [209]. These discrepancies may stem from multifaceted and dynamic nature of inflammation in this condition over time, as well from the biological complexity of the disease, cohort heterogeneity, variability in disease stages, or the impact of antipsychotic treatments and comorbid conditions. Rather than a single biomarker, TRS is likely characterized by distinct inflammatory profiles that may vary across illness stages and treatment exposures.

Overall, inflammation and immune dysregulation, appear to play an important role in TRS, with the most consistent evidence pointing to alterations in IL-6, IL-1/IL-1RA signaling, and enhanced Th17-related activity. In this context, longitudinal studies are crucial to disentangle state- from trait-related immune signatures, to capture the dynamic fluctuations of inflammatory markers across illness stages and treatment exposures, and to test their predictive value for treatment response. Beyond longitudinal designs, progress will also require larger, well-stratified cohorts.

### 4.1. The Role of Glial–Neuronal Interactions and Neurochemical Inflammation in TRS

Neuroinflammation has emerged as a central component in the pathophysiology of TRS, with increasing evidence pointing to sustained microglial activation and elevated levels of pro-inflammatory cytokines such as TNF-α, IL-1β, and IL-6 [158]. This chronic inflammatory state may contribute to persistent neuronal dysfunction, weakened BBB integrity [210], and disrupted neurotransmitter regulation within the glutamate–dopamine axis [158]. Such alterations can impair NMDAR function and exacerbate excitotoxic damage, ultimately leading to synaptic loss [211,212].

As specialized resident macrophages, microglia respond rapidly to alterations in the brain environment, mediating beneficial and detrimental effects. However, chronic activation of microglia leads to the release of pro-inflammatory cytokines, nitric oxide (NO), and pro-oxidants, contributing to neuroinflammation and neuroprogression [213,214].

In vivo PET imaging studies using the first generation tracer a [11C](R)-(1-[2-chrorophynyl]-N-methyl-N-[1-methylpropyl]-3 isoquinoline carboxamide (11C-(R)-PK11195) to map TSPO expression, as a proxy for glial activation in small subsets of patients across different illness stage, reported significant differences in regional-binding potential when they were pooled together [215]. Nonetheless, these results were not sufficiently robust to substantiate microglial activation as a consistent or defining feature of schizophrenia, at least as measured by 11C-(R)-PK11195 [216] given a high variability and susceptibility of this tracer to measurement errors. Conversely, other TSPO PET investigations, using second-generation tracers, reported decreased binding in schizophrenia [217]. Even if methodological aspects, including the choice of tracer generation and limited sample sizes introduce variability in results, we may conclude that an inflammatory phenotype may be present only in a subgroup of psychotic patients [218]. Noteworthy, a replicated finding in TSPO PET studies was a positive correlation between binding potential and age, suggesting that age-related changes may be a stronger determinant of TSPO signal than diagnostic status [216,219].

Preclinical and postmortem studies suggest that TRS is characterized by specific neuroinflammatory signatures, including increased expression of inflammasome proteins (e.g., apoptosis-associated Speck-like protein containing a card—ASC; NOD-like receptor family, pyrin domain containing 3—NLRP3; IL-18) in microglia, astrocytes, and oligodendrocytes within the frontal cortex [211]. These glial alterations may amplify immune dysregulation and interfere with synaptic integration, potentially underlying the persistent cognitive deficits observed in TRS [220]. Moreover, abnormal microglial pruning of synapses, driven by excessive immune activation, may reduce dendritic spine density and impair cortical connectivity. Excessive microglial activity may reduce spine/synapse density on cortical glutamatergic pyramidal neurons below functional thresholds, impairing network integration critical to schizophrenia pathophysiology [221,222,223]. Activated microglia mediate synaptic refinement by eliminating underutilized synapses, modulated by synaptic plasticity mechanisms such as long-term potentiation (LTP) [224]. This aligns with genetic studies linking schizophrenia to structural synaptic variations and altered NMDAR-dependent plasticity genes [211]. Excessive microglial activity may reduce spine/synapse density on cortical glutamatergic pyramidal neurons below functional thresholds, impairing network integration critical to schizophrenia pathophysiology, contributing to the pathogenesis of both negative and positive symptoms [225,226]. Microglia-driven synaptic over-pruning is further associated with gray matter loss and prefrontal-hippocampal dysfunction, underlying cognitive and negative symptoms, while cortical disinhibition may exacerbate dopaminergic hyperactivity, precipitating positive symptoms [227]. On the other hand, the synaptic loss disrupts the E/I balance, accounting for the disinhibition of mesostriatal dopaminergic projections, exacerbating dopaminergic hyperactivity, which is responsible for positive symptoms [228].

Clozapine, the only approved medication for TRS, may exert a unique neuroprotective effect against the neuroinflammation-induced damage by inhibiting microglial overactivation [229]. As demonstrated in primary cortical and mesencephalic neuron-glia cultures, clozapine exerts a neuroprotective effect against lipopolysaccharide-induced neurotoxicity by inhibiting microglial activation and reducing oxidative stress. The effect requires the presence of microglia and involves the phosphoinositide 3-kinases (PI3K) signaling pathway. In preclinical models, clozapine enhances dendritic spine formation and shows antioxidant and anti-apoptotic properties in neural stem cells exposed to glutamatergic stressors such as NMDAR antagonists [230,231]. These findings suggest that targeting neuroinflammation could be a promising therapeutic avenue, particularly in biologically defined subgroups [232,233,234]. In this regard, pharmacological strategies aimed at modulating cytokine levels or microglial activity may help preserve synaptic integrity and boost antipsychotic responsiveness in TRS. In fact, pro-inflammatory processes can alter postsynaptic density (PSD) proteins, which are critical for synaptic plasticity and NMDAR signaling [235,236,237,238]. In primary TRS, such alterations may be particularly pronounced or resistant to compensatory mechanisms, further compromising neuronal communication and contributing to cognitive deficits [12,239,240].

In summary, while preclinical and postmortem findings consistently implicate glial–neuronal dysfunction and neuroinflammation in TRS, in vivo TSPO-PET studies have yielded heterogeneous results, with age emerging as a major confounder or, alternatively, a relevant determinant. A peculiar pro-inflammatory phenotype may characterize only a subset of TRS patients. Taken together, these data indicate that glial activation contributes to treatment resistance but is unlikely to represent a universal mechanism.

### 4.2. Mitochondrial Dysfunction and Oxidative Stress in TRS

Mitochondrial dysfunction and oxidative stress are increasingly recognized as central players in the pathophysiology of TRS, intertwining with synaptic pathology, neuroinflammation, and antipsychotic response dynamics. Chronic oxidative stress disrupts intracellular Ca^2+^ homeostasis, leading to excessive neuronal Ca^2+^ influx and subsequent accumulation of ROS and reactive nitrosative species (RNS). Clinical and in vivo findings provide indirect support: patients with schizophrenia, including TRS, often display chronic oxidative stress, impaired antioxidant defenses [241], and hypothalamic–pituitary–adrenal (HPA) axis dysregulation manifesting as hypercortisolemia, which amplify lipid peroxidation and mitochondrial dysfunction [242,243,244,245].

Buosi and colleagues, investigated oxidative stress biomarkers in 89 participants, including treatment-responsive schizophrenia patients (n = 26), treatment-resistant patients (n = 27), and healthy controls (n = 36). They found increased peripheral blood levels of catalase (CAT) and malondialdehyde (MDA), and reduced superoxide dismutase (SOD) activity in patients compared to controls, but no consistent differences were observed between treatment-responsive and treatment-resistant groups, suggesting that oxidative imbalance reflects a general pathophysiological feature of schizophrenia rather than a marker of treatment resistance [241]. As is well-known, cigarette smoking generates superoxide radicals that may reduce SOD levels and contribute to higher oxidative damage, and schizophrenia population exhibits overall a greater rate of smoking compared to controls [246,247]. In another cohort CAT activity positively correlated with PANSS total scores and predicted, together with IL-6 levels, and TRS diagnosis [248].

Dysregulation of the HPA axis, often manifesting as hypercortisolemia in schizophrenia patients, amplifies oxidative damage and lipid peroxidation, further impairing mitochondrial energy metabolism [245]. Pro-inflammatory cytokines, particularly TNF-α exacerbates this cascade by disrupting astroglial glutamate reuptake, resulting in excessive extracellular glutamate, Ca^2+^-mediated excitotoxicity and neuronal apoptosis within the hippocampus [249]. These events converge on the activation of the mitogen-activated protein kinase/activator protein-1 (MAPK/AP-1) pathway, which upregulates pro-oxidant enzymes like inducible nitric oxide synthase (iNOS) and cyclooxygenase-2 (COX-2), which perpetuate ROS/RNS production [192,250,251,252]. Supporting these links, proteomic studies have identified differential expression of mitochondrial-related proteins in TRS patients [253].

Postmortem studies add more direct evidence, reporting impaired Na^+^/K^+^-ATPase (NKA) activity [254], as shown in the frontal cortex of schizophrenia patients [255], a region critical for cognition, emotional regulation, and decision-making [256,257]. NKA, a membrane-bound enzyme essential for maintaining ion gradients in the CNS, is particularly vulnerable to oxidative damage [254]. Its dysfunction has been linked to dendritic atrophy, disrupted neuronal connectivity, and glutamate excitotoxicity which are hallmarks of schizophrenia pathology [258].

The frontal cortex and hippocampus, brain regions pivotal for higher-order cognitive functions, exhibit heightened susceptibility to oxidative stress. Chronic stress models in rodents and clinical evidence reveal dendritic shrinkage, reduced synaptic plasticity, and elevated pro-inflammatory cytokines (e.g., TNF-α, IL-6) in these areas, driven by hyperactivity of the HPA axis [242,243,244].

A further in vitro study conducted on PC12 cells shows that higher clozapine concentrations exacerbate oxidative stress and cytotoxicity [259], involving the downregulation of progesterone receptor membrane component 1 (PGRMC1), a protein located on the outer membrane of mitochondria, which is implicated in maintaining mitochondrial integrity and function [260]. Clozapine has been shown to inhibit PGRMC1 activity resulting in impaired mitochondrial dynamics and increased neurotoxicity [259]. The study also points to the involvement of glucagon peptide-1 receptor (GLP-1R) and mitofusin 2 (Mfn2) in clozapine-induced mitochondrial dysfunction [259]. GLP-1R regulates mitochondrial function and dynamics by promoting the expression of Mfn2 [261], a protein essential for mitochondrial fusion and metabolism [262]. Clozapine treatment causes downregulation of both GLP-1R and Mfn2, further altering mitochondrial morphology and increasing oxidative stress [259].

Preclinical findings found that clozapine alters the activity of the adenosine monophosphate (AMP)-activated protein kinase—acetyl CoA carboxylase—carnitine palmitoyl transferase 1 (AMPK-ACC-CPT1) pathway, a central pathway of lipid metabolism, affecting the lipid composition of neuronal membranes in the rat frontal cortex [263]. Abnormalities in membrane lipid composition were also found in the frontal cortex of patients with schizophrenia [264]. In this context, beta-oxidation may potentially be linked to the pathogenesis of TRS [265]. Additionally, the study highlighted mitochondrial tryptophanyl-tRNA synthetase (WARS2), another mitochondrial protein involved in aminoacyl-tRNA synthesis [266], as causally related to TRS through colocalization analysis [152]. These findings underline the importance of mitochondrial function in maintaining neuronal health and suggest that mitochondrial process disruptions could exacerbate or underlie the treatment resistance.

Furthermore, clozapine has been shown to modulate mitochondrial complex I activity by increasing the synthesis of glutathione (GSH), a free radical scavenger, potentially mitigating oxidative damage [267]. GSH may be reduced by increased dopamine firing, as in schizophrenia, due to dopamine autoxidation. Noteworthy, GSH and NMDAR functioning are closely associated, with a reduction in GSH levels resulting in NMDAR hypofunction [268].

However, its activity may paradoxically increase ROS production [269], especially in long-term use, showing a dual role dependent on dose, duration, and receptor affinity [270,271,272]. Functional enrichment analysis performed by PWAS identified several gene ontology (GO) terms associated with mitochondria and oxidative stress [152]. Notably, “mitochondrion” (GO:0005739) emerged as one of the most significant terms enriched among the proteins linked to TRS, emphasizing the hypothesis that mitochondrial pathways are central to the TRS condition. Additionally, oxidoreductase activity (GO:0016620), which involves enzymes that mediate redox reactions, further emphasized the connection between oxidative stress mechanisms and TRS [152].

In schizophrenia, mitochondrial dysfunction is marked by altered membrane potential, impaired electron transport chain activity, and reduced adenosine triphosphate (ATP) production [273]. These abnormalities increase ROS generation, further damaging mitochondrial DNA (mtDNA), proteins, and lipids. This vicious cycle exacerbates oxidative stress and contributes to neuronal apoptosis and synaptic dysfunction [274], relevant to TRS [258]. For instance, excessive ROS production can trigger apoptotic pathways through caspase activation or disrupt mitochondrial biogenesis by harming mtDNA [275]. In the explanatory model proposed by Murray et al., MIA during pregnancy induce the release of pro-inflammatory cytokines, which elicit the overproduction of KYNA, acting as an NMDAR antagonist, and leading to NMDAR hypofunction on PV^+^ GABAergic interneurons. The resulting loss of inhibitory control enhances the firing of excitatory glutamatergic neurons projecting to the midbrain, thereby driving dopaminergic hyperactivity associated with positive symptoms. Under excitotoxic conditions, excessive glutamate release promotes free radical generation, while dopamine itself undergoes auto-oxidation, further increasing oxidative stress and tissue damage, thus amplifying the inflammatory milieu. The accumulation of ROS diminishes antioxidant defenses, lowering the availability of SOD and GSH. Reduced GSH, in turn, exacerbates NMDAR hypofunction on inhibitory interneurons, establishing a vicious cycle that sustains excitatory–inhibitory imbalance and dopaminergic dysregulation [276]. Mitochondria, as the main regulators of cellular energy and redox balance, are particularly vulnerable to this oxidative load: ROS damage mitochondrial DNA, proteins, and lipid membranes, impairing ATP production and Ca^2+^ buffering [277]. In turn, mitochondrial dysfunction exacerbates glutamatergic excitotoxicity and reduces GSH regeneration, further reinforcing NMDAR hypofunction on interneurons.

In conclusion, mitochondrial dysfunction and oxidative stress may contribute to development and persistence of excitatory/inhibitory imbalance that characterizes schizophrenia and its treatment-refractory forms.

### 4.3. Endoplasmic Reticulum Stress and Cellular Dysfunction in Schizophrenia: Relevance for Treatment Resistance

The proposed role of antipsychotics in mitigating oxidative stress may support the growing body of evidence indicating oxidative damage to DNA, carbohydrates, enzymes, proteins, and cellular lipids observed in psychiatric disorders, particularly in schizophrenia, ultimately contributing to neurodegenerative processes [278,279]. Within these processes, the endoplasmic reticulum (ER) plays a significant role, alongside mitochondria, due to its involvement in maintaining the pro-oxidant/antioxidant balance and its responsibility in the accumulation of misfolded proteins [280,281,282,283]. Furthermore, given that dopamine metabolism is a key regulator of oxidative stress, and considering hyperactivation of dopamine neurocircuits in the mesolimbic areas of patients with schizophrenia, endoplasmic reticulum stress could represent a consequence of neurotransmitter dysregulation [284,285].

The ER plays a central role in protein synthesis and modification, as well as in lipid synthesis. Under stress conditions, the ER activates a specific and evolutionarily conserved response known as the unfolded protein response (UPR). Misfolded proteins interact with molecular chaperones to repair misfolding and achieve their native conformation. When terminally misfolded, proteins are directed to the ER-associated degradation (ERAD) pathway to be removed by the ubiquitin-proteasome system [286,287].

Several studies have investigated the association between ER stress and schizophrenia due to genetic factors, including X-box-binding protein 1 (XBP-1) 116C/G and 197C/G [288,289]. In contrast, the ER stress inhibitor 4-phenylbutyric acid (4-PBA) has been suggested as a potential therapy for schizophrenia-related manifestations.

Interestingly, it is reported that olanzapine can induce hypothalamic ER stress in rodents, whereas the use of an ER stress inhibitor suppresses olanzapine-induced hyperphagia and weight gain [290]. A recent in vitro study has demonstrated that olanzapine is also able to trigger ER stress in cultured astrocytes [290]. This activation occurs before weight gain onset, indicating that astrocytic ER stress may be a contributor to antipsychotic-induced obesity [290]. This effect may be linked to antipsychotics’ metabolic disturbances, such as weight gain and obesity [290].

At a molecular level, ER stress is regulated by sensors such as protein kinase RNA-like endoplasmic reticulum kinase (PERK), activating transcription factor 6 (ATF6), and inositol-requiring enzyme 1 (IRE1), which are normally bound to the chaperone binding immunoglobulin protein/glucose-regulated protein 78 (BiP/GRP78); under stress conditions, these activate molecular pathways that influence the cellular response [291,292,293,294]. A postmortem study found altered expression of these markers in the dorsolateral prefrontal cortex of patients with schizophrenia, suggesting intrinsic dysregulation of the UPR not induced by antipsychotics [295]. Thus, based on these findings, although it is evident that markers of baseline UPR activity are abnormally expressed in schizophrenia, it remains uncertain whether these pathways react to stress in the context of chronic pharmacological exposure. Nevertheless, antipsychotics may still contribute to the exacerbation of ER stress, particularly in individuals with pre-existing molecular vulnerabilities [295].

Despite the patients in the previous study being on antipsychotics, the authors conclude that antipsychotic treatment is not sufficient to induce these changes in the brain, suggesting that observed changes are only due to the illness. Even though the evidence for ER stress in TRS is limited, these alterations may represent the link between prolonged antipsychotic exposure, impaired stress-response mechanisms, and reduced therapeutic response. Therefore, ER dysfunctions should be investigated as a potential contributor to the development and persistence of treatment resistance.

## 5. Structural Neuroimaging Biomarkers of Treatment Response and Resistance in Psychotic Disorders

Several studies have explored the relationship between structural neuroimaging features and clinical outcomes following antipsychotic treatment in patients affected by psychotic disorders. These findings exhibit a high degree of heterogeneity, largely influenced by the specific drug employed [296]. Alterations in gray matter volume, total and local gyrification, cortical sulci, and white matter tracts have been variously linked to antipsychotic resistance in first-episode psychosis (FEP) and TRS patients.

### 5.1. Gray Matter Alterations and Antipsychotic Responsiveness

In a cohort of FEP patients treated with 2 mg/day of haloperidol, greater cortical gray matter volumes were significantly associated with better responses in PANSS total, positive, and negative scores [297]. Similarly, in another sample, non-remitted FEP patients exhibited significantly lower gray matter concentration, as measured by voxel-based morphometry (VBM), in the parahippocampal gyrus bilaterally compared to responsive patients [298]. Interestingly, brain volumes correlated negatively with social withdrawal and positively with verbal memory performance [298]. Conversely, greater volumes of the right caudate, left putamen, and bilateral insula have been associated with poorer responses, as measured by PANSS total scores, following three weeks of risperidone or olanzapine treatment [299].

Differences in local gyrification were analyzed in 80 FEP patients, comparing responders, nonresponders, and healthy controls [300]. Patients exhibited significant reductions in the gyrification index across multiple regions relative to controls [300]. Notably, nonresponders showed prominent hypogyria in the bilateral insula, left frontal, and right temporal regions compared to responders [300].

Structural neuroimaging studies have identified various biomarkers associated with responsiveness or resistance to clozapine treatment in patients with TRS. A Computed Tomography (CT) study on 42 patients initiating clozapine after failing previous antipsychotic treatments found that nonresponders exhibited significantly enlarged cortical sulci at baseline, while ventricular size remained comparable between groups [301]. Multiple regression analyses further revealed that increased size of the posterior frontal and lateral temporal sulci, indicative of gray matter loss, was significantly associated with poorer treatment outcomes [301]. Another CT study on TRS patients found a significant association between prefrontal sulcal prominence (PSP) and responsiveness to six weeks of clozapine treatment, with nonresponders exhibiting the highest PSP [302].

A double-blind randomized trial investigated the effects of a 10-week treatment with haloperidol or clozapine in a cohort of TRS patients [303]. In the clozapine group, greater gray matter volume of the right prefrontal cortex was associated with less severe negative symptoms, a correlation that was not observed in the haloperidol group [303]. Moreover, in clozapine-treated patients, larger right prefrontal cortex volumes were associated with greater improvements in hostility and anxiety/depression symptoms, whereas in the haloperidol group, larger volumes correlated with higher symptom severity [303].

In a 6-month trial on 25 TRS patients undergoing magnetic resonance (MRI) and ^18^F-deoxyglucose Positron Emission Tomography (^18^FDG-PET) scans before and after clozapine treatment, greater dorsolateral prefrontal cortex volume predicted negative symptom reduction, while positive symptom response was linked to greater temporal gray-matter volumes [304]. Otherwise, disorganization symptom improvement was inversely related to total intracranial and hippocampal volume [304].

A cross-sectional MRI study found that TRS patients exhibited significant gray matter volume loss in the temporal gyri (superior, middle, and inferior part), pre- and postcentral gyri, superior and middle frontal gyri, right supramarginal gyrus, and right lateral occipital cortex compared to individuals responsive to first-line antipsychotics [305]. Additionally, UTRS patients showed reduced gray matter volume in the right parietal operculum and left cerebellum compared with responders, although no significant differences emerged between TRS and UTRS groups [305]. A direct comparison between TRS and UTRS patients further revealed greater cortical thinning in the left medial frontal cortex and right middle temporal cortex in UTRS patients 6–9 months after clozapine initiation [306], supporting evidence of hypofrontality in clozapine-resistant patients [307].

Finally, an intrinsic cortical curvature (ICC) study in a cohort of 38 first-line responders, 30 clozapine-resistant TRS, 37 clozapine-responsive TRS, and 52 healthy controls revealed that both TRS groups exhibited increased ICC in the dorsolateral prefrontal cortex and temporal structures, while clozapine-resistant TRS patients also showed ICC elevations in the left ACC [308].

### 5.2. White Matter Integrity and Treatment Response in Psychotic Disorders: Insights from Diffusion Tensor Imaging

Using MRI with diffusion tensor imaging (DTI), increased diffusivity, indicative of myelin integrity impairment, was observed in treatment responders but not in treatment-resistant patients, compared to controls [309]. Notably, antipsychotic treatment led to an 84% improvement in myelin integrity among responders, while no significant changes occurred in treatment-resistant individuals [309].

Further, DTI tractography was used to measure fractional anisotropy (FA) in white matter tracts connecting frontal and temporal regions in 44 FEP patients, categorized by poor or good response following a 6-month antipsychotic trial [310]. In contrast, FA was reduced in the uncinate and superior longitudinal fasciculi in FEP patients compared to controls, with greater white matter alterations in patients with poor outcomes [310].

A longitudinal study found that abnormalities in fiber tracts, particularly the inferior fronto-occipital fasciculus and the superior longitudinal fasciculus, significantly predicted individual responses to antipsychotic treatment [311]. Similarly, in a 12-week prospective study on FEP patients, lower FA at baseline, especially in the uncinate, cingulum, and corpus callosum, was associated with poor treatment outcomes, while responders exhibited FA values comparable to healthy controls [312]. Although FA increased in both groups after 12 weeks, differences between responders and non-responders persisted, albeit to a lesser extent [312].

In a more recent cross-sectional study, patients with TRS exhibited lower FA than healthy controls in multiple tracts, including the superior longitudinal fasciculus, corpus callosum, thalamic radiation, corticospinal tract, internal capsule, corona radiata, and fronto-occipital fasciculus [313]. Furthermore, FA reductions in the superior longitudinal fasciculus distinguished TRS from UTRS [313].

Overall, these findings highlight the complex relationship between structural neuroimaging features and clinical outcomes in psychotic disorders. The variability across different antipsychotics emphasizes the need for personalized treatment strategies based on individual neurobiological profiles. Of interest, alterations in the frontal and temporal lobes, as well as in the white matter tracts connecting these regions, before the onset of antipsychotic treatment, may negatively impact responsiveness to clozapine, potentially contributing to ultra-resistance phenotypes.

### 5.3. Alterations in Functional Connectivity and Network Parameters in Antipsychotic-Refractory Patients: Insights from fMRI Studies

Several functional neuroimaging studies using blood-oxygenation-level-dependent functional magnetic resonance imaging (BOLD-fMRI) have revealed widespread connectivity alterations in TRS patients, suggesting disruptions in large-scale brain networks associated with poor responses to antipsychotic treatment [314,315]. Further, multiple longitudinal studies have analyzed brain functional characteristics related to treatment response in FEP patients.

#### 5.3.1. Alterations in the Functional Connectivity of Cortical Regions as Predictors of Antipsychotic Resistance: Evidence from Resting State fMRI

In a cohort of schizophrenia patients with medication-resistant auditory-verbal hallucinations, reduced functional connectivity of the temporoparietal junction was observed, particularly in its connection with Broca’s area [316]. Moreover, the severity of hallucinations was negatively correlated with functional connectivity between the left temporoparietal junction and both the left and right anterior cingulate, as well as the left and right amygdala [316]. Similarly, patients with schizophrenia who had persistent and treatment-refractory auditory-verbal hallucinations exhibited dysconnectivity in bilateral temporal regions and cingulate cortex compared to healthy controls [317]. The severity of auditory hallucinations correlated with functional connectivity of the left anterior cingulate, left superior temporal gyrus, and right lateral prefrontal cortex [317].

Cross-network abnormalities between the Default-Mode Network (DMN) and the salience system in patients with persistent hallucinations have been proposed [318]. Specifically, patients with chronic hallucinations resistant to pharmacological treatment exhibited higher functional connectivity between the dorsomedial prefrontal cortex and bilateral central opercular cortex, bilateral insular cortex, and bilateral precentral gyrus compared to patients without hallucinations and healthy controls [318]. TRS patients also exhibited lower functional connectivity between the ventromedial prefrontal cortex and bilateral paracingulate and dorsal ACC [318]. Alterations in the functional connectivity of the ACC with the cuneus and thalamus were also observed in the comparison between TRS patients and individuals responsive to antipsychotics [319].

In a recent study conducted on 294 patients, abnormal frontal-limbic, frontal-parietal, and occipital-temporal functional connectivity was effective in distinguishing treatment-resistant patients from treatment-responsive subjects, and further correlated with disease progression, symptoms, and medication dosage [320]. Reductions in network global parameters, such as global strength and efficiency, and decreased edges, especially in frontotemporal, fronto-occipital, and temporo-occipital connections, were also observed in TRS patients compared to controls [321]. Alterations in network parameters, including cerebellar-frontal, cingulo-fronto-temporal, and frontoparietal disconnections, were further detected in UTRS subjects compared to healthy controls, with patients exhibiting the weakest connection strength [322].

In a cohort of 23 individuals affected by schizophrenia compared to 32 matched controls, a pattern of aberrant network integration and segregation, such as reduced global efficiency and increased clustering coefficient, was detected and efficiently modulated by 6 weeks of risperidone only in those who responded to treatment [323]. Conversely, decreased clustering coefficient was found in FEP patients, and its increase, modulated by antipsychotic treatment, was associated with the improvement of negative symptoms [324].

In a cohort of 42 FEP patients treated for 8 weeks with risperidone, functional connectivity within the DMN, especially between the posterior cingulate/precuneus and the prefrontal cortex, correlated with an improvement in positive symptoms [325]. Another study employing random forest analysis showed that the connectivity of the hippocampus with the insular–opercular cortex, superior frontal gyrus, precentral gyrus, and postcentral gyrus predicted treatment response with an area under the curve (AUC) of 0.95 [326]. Consistently, in a 12-month longitudinal study, higher functional connectivity within a network including the left hippocampus, bilateral insula, and temporal poles was found as a predictor of treatment resistance [327].

Patients non-responding to first-line antipsychotics and eligible for clozapine treatment exhibited enhanced functional connectivity within the sensorimotor network and precuneus compared to drug responders [328]. Disrupted functional connectivity in the sensory-motor network was also observed in 60 FEP patients with drug-naïve schizophrenia and predicted the improvement of positive scores after medication [329].

#### 5.3.2. Striatal Connectivity and Treatment Response Prediction

In a FEP cohort, a striatal connectivity index was created by considering functional connections of the striatum with other brain regions and then was tested on 40 newly hospitalized chronic patients with acute psychosis, showing 80% sensitivity and 75% specificity for the prediction of resistance/responsiveness to antipsychotics [330]. Functional striatal abnormalities proved to be effective in predicting antipsychotic treatment response also in a larger sample (1100 individuals) of schizophrenia patients [331]. In drug-naïve FEP patients treated with risperidone monotherapy for 8 weeks, the dorsal striatal pathway at baseline could predict negative symptom improvement in patients, while ventral striatal pathways could predict positive symptom improvement [332]. Consistently, dorsal striatal hypoconnectivity was effective in predicting responses to 6 weeks of risperidone treatment in unmedicated FEP patients [333].

In a sample of 38 individuals with schizophrenia stratified by refractory and non-refractory patients, TRS subjects exhibited reduced connectivity between the ventral striatum and substantia nigra, as well as more severe disturbances of corticostriatal connectivity [334]. In a longitudinal study exploring the effect of 12 weeks of clozapine treatment, responses were associated with an increase in corticostriatal connectivity between the dorsal caudate and regions of the frontoparietal network [335].

#### 5.3.3. Functional Connectivity Alterations in Cognitive Task fMRI Studies

Disruptions in functional connectivity linked to antipsychotic resistance also emerged in fMRI studies employing cognitive tasks. In a longitudinal study conducted on male schizophrenia patients, a reduced signal change in the dorsolateral prefrontal cortex at baseline, measured as the difference in signal between novel and practiced working-memory tasks, was associated with poor treatment responses at 10 weeks [336]. During visuospatial tests, schizophrenia patients stabilized with atypical antipsychotics other than clozapine showed significant activations in temporal and occipital regions, which were not detected in TRS patients treated with clozapine [337]. After the employment of emotionally positive, negative, and neutral images, hyperactivations were observed in the dorsolateral prefrontal cortex, left cerebellum, and cingulate cortex of TRS patients treated with clozapine compared to patients with adequate response to antipsychotics other than clozapine and healthy controls [338]. In a cognitive task fMRI study, greater functional connectivity between the ACC and bilateral putamen at the baseline predicted subsequently a better treatment response to a 6-week trial with risperidone [339].

Although heterogeneous, these results highlight differences in the functional connectivity profile, as detected by fMRI, in TRS patients compared to both antipsychotic-responsive subjects and healthy controls, especially in the prefrontal, temporal, and ACC. Further alterations were observed in subcortical-cortical pathways and global network parameters, often but not always pointing to reduced functional connectivity in antipsychotic-refractory patients. Interestingly, first-line treatment response, especially to risperidone, was effectively predicted by the functional connectivity of subcortical nodes.

### 5.4. Metabolic Activity in Treatment-Resistant Schizophrenia: Evidence Collected from ^18^F-Deoxyglucose Positron Emission Tomography

Relatively few studies have investigated the metabolic characteristics of antipsychotic resistance in schizophrenia by FDG-PET.

A study by Molina et al. examined 25 TRS patients using structural MRI and FDG-PET, both before and after clozapine treatment [304]. Notably, high baseline cortical volume and metabolic activity in the dorsolateral prefrontal cortex significantly predicted improvement in negative symptoms following clozapine therapy.

In a case series, Roldan et al. investigated the effects of deep brain stimulation (DBS) targeting the subgenual ACC or the nucleus accumbens in TRS patients [340]. FDG-PET scans, conducted at baseline and after at least six months of stimulation, revealed that DBS-induced changes in glucose metabolism were associated with clinical improvement. Specifically, altered metabolic activity was observed in the medial prefrontal cortex, dorsolateral prefrontal cortex, ACC, caudate nucleus, nucleus accumbens core, hippocampus, and thalamus.

In a study performed by our group, 41 patients and 12 controls were investigated by FDG-PET. Areas of significant bilateral hypometabolism in the superior frontal gyrus were found in TRS compared to individuals responsive to antipsychotics. Moreover, reduced parietal and frontal metabolism was associated with high PANSS disorganization factor scores in TRS patients. Significant hypermetabolism was observed in the temporo-occipital regions in TRS compared to antipsychotic responsive patients and controls [341].

Recently, our group conducted a metabolic connectivity analysis using FDG-PET data, revealing key differences in neuronal metabolism between TRS patients and controls. TRS patients exhibited globally reduced connectivity, with prominent alterations in the frontal regions, DMN, and dorsal dopamine pathway. Additionally, specific disruptions in connectivity between the frontal and temporal regions, hippocampus, and cingulate cortex, as well as in the supplementary motor area and subcortical-cortical pathways, distinguished treatment-responsive individuals from those who were refractory to antipsychotic treatment [342].

Overall, FDG-PET studies suggest that the most affected brain regions in TRS are the frontal cortex, especially the dorsolateral prefrontal cortex, and the ACC. These regions may play a crucial role in both the pathophysiology of treatment resistance and the response to clozapine, as evidenced by baseline metabolic alterations associated with symptom improvement following treatment. Despite these insights, PET studies on antipsychotic resistance remain relatively scarce compared to the extensive body of research using fMRI. Expanding PET studies could provide a deeper understanding of the biological basis of treatment resistance by offering a more direct measure of neuronal activity, with higher validity and replicability than fMRI applications.

Taken together, structural, functional, and metabolic neuroimaging evidence converges on the critical function of brain integrity and network structure in the modulation of the response to antipsychotic treatment. Throughout the broad spectrum of neuroimaging techniques, alterations in frontotemporal circuits, corticostriatal networks, and the ACC seem to be the most implicated as potential treatment resistance biomarkers. These findings complement a transdiagnostic framework of antipsychotic non-responsiveness and emphasize the need for multimodal, integrative imaging approaches to guide personalized treatment interventions and maximize clinical outcomes in psychotic disorders.

## 6. Discussion

TRS is a highly relevant mental health issue considering both its epidemiological impact, affecting about one third of schizophrenia patients, and its significant effect on disease trajectory, particularly in terms of severely impaired functioning [1,2].

Therefore, trying to dissect the potential divergent neurobiology of TRS patients compared to schizophrenia-responsive ones, could be of great significance. Such insights could enhance early diagnostic accuracy, promote timely initiation of appropriate therapies—especially considering that clozapine introduction is often delayed by several years—and inform the development of novel pharmacological and non-pharmacological treatment strategies. In Table 1, we have summarized the proposed main differences among TRS and non-TRS. A major stratification between responders and TRS patients could be represented by the differential role and ‘weight’ of dopamine dysfunction. It should be underlined that divergent dopamine dysfunction between responders and resistant patients should not be limited to the apparent lack of increased striatal dopamine uptake in the resistant ones [343] and may encompass other relevant mechanisms above all different dopamine functional activity at presynaptic side [344]. However, is the putative divergent dopaminergic dynamics necessary and sufficient to separate treatment-resistant patients from treatment-responsive ones? Probably not, and other factors should be taken in the account for this difference in the outcome after antipsychotic treatment.

Among multiple neurotransmitter systems, glutamate signaling is the one that has attracted more attention in TRS pathophysiology. Some of the most compelling, though still limited, evidence for glutamatergic alterations in TRS comes from in vivo ^1^H-MRS studies [345,346,347]. However, it is difficult to draw a definitive conclusion considering the small number of patients enrolled in the studies over the time. One different perspective on glutamate involvement in TRS emerges from ^18^FGD PET studies, which have shown the possibility to stratify patients responsive and not responsive to antipsychotics based on the brain region specific metabolism. It has been proposed that ^18^FGD uptake could represent a possible proxy of glutamate metabolism based on the role of glucose in the synthesis of alpha-ketoglutarate, a major precursor of glutamate [348,349,350,351].

Furthermore, the interest for glutamatergic alteration in TRS stems also from the potential role of D-amino acids and related biosynthetic enzymes in the pathophysiology and possibly in the treatment of TRS and UTRS conditions [40,41]. More studies and trials are needed and warranted to reach a clear-cut framework of how and to what extent D-amino acids may be efficacious at bypassing the lack of response to antipsychotic treatment.

The role of inflammation and oxidative stress in TRS neurobiology is at the beginning of the investigation and remains a matter of debate, as it is more broadly in schizophrenia pathophysiology beyond treatment response. Therefore, a prudent and critical approach is needed. Nevertheless, emerging findings on the complex interplay between neuroinflammation, neurotransmitters’ regulation, and microglial activity deserve attention. These insights underscore the need for further studies at both preclinical and clinical levels to explore the potential role of agents acting on inflammation or oxidative stress in combination with antipsychotics in TRS [352,353,354].

Genetics, neuroimaging, and imaging-genetics approaches have significantly contributed to the identification of candidate genes potentially involved in treatment resistance, while also demonstrating, through multimodal imaging techniques (including MRI, fMRI, MR spectroscopy—MRS—and PET), their utility in stratifying patients according to antipsychotic treatment response. A promising strategy in schizophrenia research is to tackle one of the quintessential features of schizophrenia: the aberrant brain connectivity that may be further pronounced in patients who do not respond to antipsychotics.

Artificial Intelligence (AI) applied to radiomics [355] has emerged as a powerful tool for extracting clinically relevant information from imaging datasets, potentially aiding in the stratification of patients based on treatment response [356]. Currently, the most feasible AI application is represented mainly by machine learning algorithms, with most existing studies focusing on brain morphometry and functional connectivity independently of treatment outcomes [357]. Nonetheless, recent findings suggest that machine learning-based analysis of brain connectivity may also provide valuable insights specific to TRS [342] and more studies leveraging this strategy are warranted.

**Table 1:** Summary of main differences between treatment-resistant and treatment-responsive schizophrenia patients. TRS = treatment-resistant schizophrenia; SCZ = schizophrenia, responsive to treatment; PRL = prolactin; APS = antipsychotics; D2R = Dopamine receptor 2; Glu = glutamate; Glx = glutamate and glutamine concentration; ACC = anterior cingulate cortex; E/I = excitatory/inhibitory; GSH = glutathione; UTRS = ultra-treatment-resistant schizophrenia; PRS = polygenic risk score; CNVs = copy number variants.

	TRS	SCZ
Dopamine	-Poor clinical response with APS treatment despite adequate D2R occupancy; -Possibly lower PRL levels, suggesting non-dopaminergic mechanisms;	-Central dopaminergic dysfunction with striatal hyperdopaminergia; -Symptoms generally improve with D_2_ blockade; -Prolactin elevation consistent with dopaminergic antagonism;
Glutamate/GABA	-Stronger glutammatergic dysfunctions; -elevated Glu/Glx in ACC; -NMDA hypofunction; -E/I imbalance with reduced GABAergic inhibition	-Cortical glutamatergic dysfunction (↑ Glu/Glx in striatum and medial temporal lobe); -subtle NMDA hypofunction; -mild GABAergic alterations;
Inflammation and immunity	-↑ IL-6, IL-1RA, activated monocytes; -↓ anti-inflammatory proteins (e.g., CC16); -in UTRS: ↑ IL-6, TNF-α, IFNγ (Th17 pathway)	-Inflammatory alterations less pronounced; -Occasional ↑ IL-6 or ↑ CRP; -Microglial alterations occasionally reported;
Oxidative stress	-Stronger evidence of oxidative stress; -↓ GSH, mitochondrial dysfunction	-Evidence of oxidative stress (↑ lipid peroxidation, ↓ antioxidant defenses); -GSH levels often preserved;
Genomics/Pharmacogenomics	-Higher impact of metabolic polymorphisms (*CYP2D6*, *CYP1A2*, *CYP3A4*, COMT Val158Met, transporters *ABCB1*, *ABCC2*); -Higher association with glutamatergic and GABAergic genes variants; -Higher cumulative PRS burden than SCZ; -CNVs enriched in TRS;	– Enrichment for common SCZ risk variants (e.g., DRD2, COMT Val158Met, GRM3); – PRS indicates increased liability to psychosis compared with general population; – Rare but high risk CNVs (e.g., 22q11.2, 15q13.3, NRXN1 delections) contribute to SCZ risk;
Neuroimaging	-Greater cortical thinning; -↑ Glu in anterior cingulate cortex; -More marked connectivity abnormalities	-Cortical thinning and connectivity alterations present but less severe; -Abnormal striatal dopamine synthesis

## 7. Conclusions and Future Perspectives

**Figure 2:**
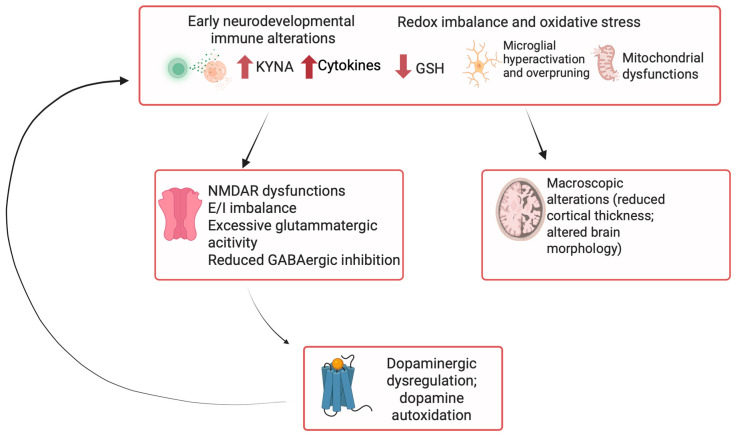
Schematic representation of the proposed pathophysiological cycle underlying treatment-resistant schizophrenia. KYNA = kynurenic acid; E/I = excitatory/inhibitory; GSH = glutathione; NMDAR = N-methyl-D-aspartate receptor; GABA = γ-aminobutyric acid. Created with Biorender.com.

A unifying mechanistic map may be helpful in terms of guiding interpretations of the presented data. Within such a speculative model (Figure 2), early neurodevelopmental immune perturbations could initiate a long-lasting vulnerability. These early events may trigger (i) a systemic and central inflammatory response, setting a pro-inflammatory milieu that includes disturbances of the KP and overproduction of KYNA; and (ii) redox imbalance and oxidative stress, which further promote mitochondrial alterations, microglial overactivation, excessive synaptic pruning, and progressive loss of connectivity (and in turn, macroscopic alterations in brain morphology, such as changes in cortical thickness). Elevated KYNA levels may be responsible for NMDAR hypofunction; similarly, GSH reduction acts in the same direction, further impairing NMDAR activity. Together, these processes converge on cortical E/I imbalance, characterized by excessive glutamatergic drive and impaired GABAergic inhibition, which eventually cascade into downstream dopaminergic dysregulation. Dopamine hyperactivity may then reinforce the cycle by generating additional reactive species due to dopamine autoxidation, thereby maintaining a state of neuroprogression.

Each of the biological findings reviewed here can be plausibly located within this cascade. For instance, increased cytokine levels can be viewed as initial drivers of vulnerability; in parallel, KP abnormalities and reduced antioxidant defenses represent the intermediate link from early events to NMDAR hypofunction, while dopaminergic hyperactivity marks the downstream expression of the disorder. Within this picture, primary TRS may arise from early immune, redox, and glutamatergic disruptions with relatively normal presynaptic dopamine synthesis. Antipsychotic treatment may add an additional “step”, inducing receptor adaptation and sensitization typical of secondary TRS. Although still a speculative hypothesis, clozapine-resistant or UTRS may embody the cumulative failure of multiple systems described above, probably compounded by other yet unidentified mechanisms.

## Abbreviations

TRS	treatment-resistant schizophrenia
PANSS	positive and negative symptoms scale
TRRIP	treatment response and resistance in psychosis
DSP	dopamine supersensitivity psychosis
UTRS	ultra-treatment-resistant schizophrenia
ECT	electroconvulsive therapy
D2R	dopamine D2 receptor
D3R	dopamine D3 receptor
NMDAR	N-methyl-D-aspartate receptor
^1^H-MRS	proton magnetic resonance spectroscopy
Glx	glutamate+glutamine
SPECT	single-photon emission computed tomography
GlyT1	glycine transporter 1
GABA	γ-aminobutyric acid
GABA-A	γ-aminobutyric acid receptor A
GABA-B	γ-aminobutyric acid receptor B
DAO	D-amino acid oxidase
E/I	excitatory/inhibitory
TMS	transcranial magnetic stimulation
SICI	short-interval intracortical inhibition
EMG	electromyography
nAChRs	nicotinic acetylcholine receptors
mAChRs	muscarinic acetylcholine receptors
CHRNA7	cholinergic receptor nicotinic alpha 7 subunit
PET	positron emission tomography
TSPO	18 kDa translocator protein
AMPAR	alpha-amino-3-hydroxy-5-methyl-4-isoxazolepropionic acid receptor
PAMs	positive allosteric modulators
Ca^2+^	Calcium
M1	Muscarinic receptor 1
M2	Muscarinic receptor 2
M3	Muscarinic receptor 3
M4	Muscarinic receptor 4
M5	Muscarinic receptor 5
BBB	blood–brain barrier
US	United States
FDA	Food and Drug Administration
CSF	cerebrospinal fluid
MHPG	3-methoxy-4-hydroxyphenylglycol
α1	alpha-1 adrenergic receptor
VTA	ventral tegmental area
GWAS	genome-wide association study
DDC	aromatic L-amino acid decarboxylase
L-DOPA	3,4-dihydroxyphenylalanine
5-HTP	5-hydroxytryptophan
GRM3	metabotropic glutamate receptor 3 gene
mGluR3	metabotropic glutamate receptor 3
GAD1	glutamate decarboxylase 1
GABBR2	gamma-aminobutyric acid type B receptor subunit 2
DRD1	dopamine D1 receptor gene
DRD2	dopamine D2 receptor gene
OXT	oxytocin
BDNF	brain-derived neurotrophic factor
SNP	single-nucleotide polymorphism
HTR2A	5-hydroxytryptamine receptor 2A gene
HTR2C	5-hydroxytryptamine receptor 2C gene
SLC6A4	solute carrier family 6 member 4
5-HTTLPR	serotonin-transporter-linked promoter region
*CYP2D6*	*Cytochrome P450 2D6*
*CYP1A2*	*Cytochrome P450 1A2*
*CYP3A4*	*Cytochrome P450 3A4*
*CYP2C9*	*Cytochrome P450 2C49*
COMT	catechol-O-methyltransferase
*SLC22A1*	*Solute Carrier Family 22 Member 1*
*OCT*	*organic cation transporter 1*
*ABCB1*	*ATP-binding cassette subfamily B member 1*
*MRP2*	*multidrug resistance-associated protein 2*
*ABCC2*	*ATP-binding cassette subfamily C member 2 protein*
APOC3	Apolipoprotein C-III
CNVs	Copy Number Variations
PRS	polygenic risk score
PWAS	proteome-wide association studies
CPT2	carnitine palmitoyl transferases 2
ApoL2	apolipoprotein L2
PRDX1	Peroxiredoxin 1
KP	kynurenine pathway
KYNA	Kynurenic acid
QUIN	quinolinic acid
ROS	Reactive oxygen species
GAD	glutamic acid decarboxylase
SHMT2	serine hydroxymethyltransferase
PCP	phencyclidine
DAT	dopamine transporter
NR1	N-methyl-D-aspartate (NMDA) receptor 1 subunit
vGLUT1	vesicular glutamate transporter 1
ACC	anterior cingulate cortex
MIA	maternal immune activation
TNF-α	tumor necrosis factor
sIL-6R	IL-6 soluble receptor
IL-1β	interleukins1β
IL-6	interleukins6
IL-6-R	Interleukin-6 receptor
CC16	Clara cell protein 16
IFN-γ	interferon-γ
IL-1RA	IL-1 receptor antagonist
IL-12/IL-23p40	Interleukin-12/Interleukin-23 subunit p40
IL-17A	Interleukin-17A
IL-10	Interleukin-10
B2M	beta-2 microglobulin
CRP	C-reactive protein
IL-8	interleukins8
Th17	T helper 17 cells
ASC	apoptosis-associated speck-like protein containing a CARD (an adaptor protein essential for inflammasome assembly)
NLRP3	NOD-like receptor family, pyrin domain containing 3
IL-8	interleukins18
LTP	long-term potentiation
PI3K	Phosphoinositide 3-kinases
RNS	reactive nitrosative species
HPA	hypothalamic–pituitary–adrenal
CAT	catalase
SOD	superoxide dismutase
MDA	malondialdehyde
MAPK	mitogen-activated protein kinase
AP-1	Activator Protein-1
iNOS	inducible nitric oxide synthase
COX-2	cyclooxygenase-2
NKA	Na^+^/K^+^-ATPase
CNS	central nervous system
PGRMC1	progesterone receptor membrane component 1
GLP-1R	glucagon peptide-1 receptor
Mfn2	mitofusin 2
AMP	adenosine monophosphate
AMPK-ACC-CPT1 pathway	AMP-activated protein kinase-acetyl CoA carboxylase-carnitine palmitoyl transferase 1 pathway
WARS2	mitochondrial tryptophanyl-tRNA synthetase
GSH	glutathione
GO	gene ontology
mtDNA	mitochondrial DNA
PV^+^	parvalbumin positive
ATP	adenosine triphosphate
ER	endoplasmic reticulum
UPR	unfolded protein response
ERAD	ER-associated degradation
XBP-1	X-box-binding protein 1
4-PBA	4-phenylbutyric acid
PERK	protein kinase RNA-like endoplasmic reticulum kinase
ATF6	activating transcription factor 6
IRE1	Inositol-requiring enzyme 1
BiP/GRP78	binding immunoglobulin protein/glucose-regulated protein 78
FEP	first-episode psychosis
VBM	voxel-based morphometry
CT	computed tomography
PSP	prefrontal sulcal prominence
^18^FDG-PET	^18^F-deoxyglucose positron emission tomography
MRI	magnetic resonance imaging
ICC	intrinsic cortical curvature
DTI	diffusion tensor imaging
FA	fractional anisotropy
BOLD-fMRI	blood-oxygenation-level-dependent functional magnetic resonance imaging
DMN	default-mode network
AUC	area under the curve
fMRI	functional magnetic resonance imaging
DBS	deep brain stimulation
AI	artificial intelligence
MRS	magnetic resonance spectroscopy
